# The development of a standardised diet history tool to support the diagnosis of food allergy

**DOI:** 10.1186/s13601-015-0050-2

**Published:** 2015-02-19

**Authors:** Isabel J Skypala, Carina Venter, Rosan Meyer, Nicolette W deJong, Adam T Fox, Marion Groetch, J N Oude Elberink, Aline Sprikkelman, Louiza Diamandi, Berber J Vlieg-Boerstra, the Allergy-focussed Diet History Task Force of the European Academy of Allergy and Clinical Immunology

**Affiliations:** Royal Brompton & Harefield NHS Foundation Trust, London, UK; The David Hide 6. Asthma and Allergy Research Centre, Isle of Wight, UK; Great Ormond Street NHS Foundation Trust, London, UK; Erasmus MC, Rotterdam, NL Netherlands; Guy’s and St Thomas’ Hospitals NHS Foundation Trust and Kings College, London, UK; Jaffe Food Allergy Institute, Icahn School of Medicine at Mount Sinai, New York, USA; Department of Allergology, University Medical Centre of Groningen, University of Groningen, Groningen, NL Netherlands; Emma Children’s Hospital Academic Medical Centre, Amsterdam, NL Netherlands; University of Athens, Athens, GR Greece; Emma Children’s Hospital, Paediatric Respiratory Medicine and Allergy, Academic Medical Centre, University of Amsterdam, Amsterdam, NL Netherlands

**Keywords:** History, Diet, Tool, Allergy, Food, Diagnosis

## Abstract

The disparity between reported and diagnosed food allergy makes robust diagnosis imperative. The allergy-focussed history is an important starting point, but published literature on its efficacy is sparse. Using a structured approach to connect symptoms, suspected foods and dietary intake, a multi-disciplinary task force of the European Academy of Allergy and Clinical Immunology developed paediatric and adult diet history tools. Both tools are divided into stages using traffic light labelling (red, amber and green). The red stage requires the practitioner to gather relevant information on symptoms, atopic history, food triggers, foods eaten and nutritional issues. The amber stage facilitates interpretation of the responses to the red-stage questions, thus enabling the practitioner to prepare to move forward. The final green stage provides a summary template and test algorithm to support continuation down the diagnostic pathway. These tools will provide a standardised, practical approach to support food allergy diagnosis, ensuring that all relevant information is captured and interpreted in a robust manner. Future work is required to validate their use in diverse age groups, disease entities and in different countries, in order to account for differences in health care systems, food availability and dietary norms.

**Electronic supplementary material:**

The online version of this article (doi:10.1186/s13601-015-0050-2) contains supplementary material, which is available to authorized users.

## Introduction

Adverse reactions to foods are frequently reported, however only those involving immunological mechanisms, including both immunoglobulin E (IgE) and non-IgE mediated, can be defined as food allergy (FA) [1]. The prevalence of FA varies worldwide, but the rate of true FA in children and adults is consistently lower than self-reported rates [2-5]. A European systematic review found the overall point prevalence in Europe of self-reported FA to be 5.9% (95% CI: 5.7-6.1), compared with a food challenge confirmed FA rate of 0.9% (95% CI: 0.8-1.1) [6]. Reported FA persists over time in adults [7], and age-related changes can affect the immune system, increasing the potential for newly diagnosed food allergy in older adults [8].

This disparity between reported and actual levels of FA make a robust diagnosis imperative in order to avoid overt and/or unnecessary dietary restrictions leading to either continuation of symptoms due to the wrong food being eliminated or nutritional deficiencies [9,10]. Guidelines [1,11-13] provide expert evidence-based support for the diagnosis of FA, which includes taking an allergy focussed history, performing appropriate tests and food reintroduction or controlled food challenge. Although international consensus agrees that the allergy-focussed history is a key part of the diagnostic pathway [1,12], the lack of standardisation, and variable expertise of the history taker, may prevent the ascertainment of sufficient information, leading to poor interpretation of the facts.

However, when the right questions are posed and the answers are systematically linked to appropriate actions, an allergy history can be invaluable [11,14], and for some conditions, history has been demonstrated to be wholly diagnostic [15]. Included in the additional supporting information of recent European Academy of Allergy and Clinical Immunology (EAACI) FA guidelines was a useful list of key questions for the clinical history [1] Therefore these guidelines not suggest an allergy-focussed history is fundamental to the establishment of a diagnosis, they also provide a starting point for a structured approach to history-taking. Recognition of the importance of the diet and clinical history by EAACI led to the formation of an EAACI Task Force to develop paediatric and adult diet history tools for use by health care professionals.

## Methods

The Allergy-focussed Diet History Task Force, an international multi-professional group of food allergy experts, agreed that evidence-based tools would need to be developed. In order to gather evidence of links between reported symptoms, trigger foods and dietary intake, and the existence of any published diagnostic questionnaires, a search on Pub Med and Medline was undertaken. The search terms shown in Table 1 were used, and papers from 1990 to January 2012 were selected in the order given below:

1. Randomized controlled trials
2. Non-randomized controlled clinical trials
3. Before and after clinical trials
4. Prevalence studies employing oral food challenge
5. Systematic reviews and other meta analyses
6. Observational studies – cohort or case reports
7. Other subject reviews

**Table 1:** **Search terms for review**

Tree	food hypersensitivities
Tree	food hypersensitivity
Tree	hypersensitivity food
allergies food	questionnaire
allergy food	questionnaires
assessing	recognition
assessment	recognise
detect	recognising
detecting	screen
detection	screening
diagnose	signs
diagnoses	symptoms
diagnosing	work up
diagnosis	workup
diagnostic intent	
evaluating	
evaluation	
food allergies	
food allergy	

The search resulted in 36 publications, but only one study measured the diagnostic efficacy of history against validated test methods [15]. Access to more rigorous methodology employed by the EAACI Food Allergy Guidelines Task Force [6], yielded no additional appropriate papers. In the absence of a lack of suitable published evidence, the Task Force undertook to develop age-specific diet history tools based on expert opinion, with the factors considered being supported by guidelines or underpinned by evidence from individual studies. A structure for the tools was established to enable rapid assimilation and interpretation of answers to standard questions on symptoms, atopic history, family history of atopy, co-factors, suspected or known food triggers, quantity of food involved, foods currently consumed and avoided, cross-reacting foods, growth and development and likelihood of nutritional risk. The pooled knowledge and expertise of the panel greatly contributed to the construction of the questions, especially where evidence was lacking.

## Results

Two allergy-focussed diet history tools for paediatric and adult patients were developed (see Additional files 1 and 2) based on a traffic light scheme similar to those used to inform consumers about healthy eating or energy efficiency:

Red - gather information through questioning the patient on their allergic history, foods habitually eaten, nutritional status, and relevant co-factors.

Amber – facilitates interpretation of the responses to the red-stage questions by linking the information gained to match potential diagnostic pathways, utilising the algorithms and tables provided to refine and formulate a potential diagnosis thus enabling the practitioner to prepare to move forward.

Green - the way ahead is clear to institute appropriate testing and/or onward referral to a specialist allergy centre, gastroenterologist or a specialist dietitian.

## Red stage - questioning and linking

### Symptoms and atopic history

#### Symptoms

The characterization of symptoms is an essential first step when taking a history from someone presenting with adverse reactions to foods.

#### Presenting symptoms: IgE-mediated FA

A number of guidelines have summarised these symptoms [1,11-13], primarily those seen in children, which can be skin related (pruritus, erythema, acute urticaria, acute angioedema), gastro-intestinal (anal pruritus, colicky abdominal pain, diarrhoea and vomiting) or upper and lower respiratory symptoms. In adults, skin reactions are also associated with IgE-mediated food allergy [16], but gastrointestinal symptoms are predominant especially dysaesthesia of the tongue, oro-pharyngeal pruritus and vomiting [17-20]. Rhinitis, conjunctivitis and asthma are also presenting features of adult food allergy [5,15] Tachycardia, hypotension, throat tightness, bronchospasm, laryngeal oedema, shortness of breath, syncope and anaphylaxis can affect all ages [21,22].

#### Presenting symptoms: non-IgE mediated FA

Proctocolitis [23] is characterised by blood and mucous in the stools and occasional diarrhoea, although the infant appears well. The symptoms of food protein-induced enterocolitis syndrome (FPIES) include late onset gastro-intestinal symptoms of profuse vomiting, diarrhoea, pallor and occasionally hypovolemic shock [24]. Children and infants with Eosinophilic Oesophagitis (EoE) often show signs of chronic vomiting, abdominal pain, poor appetite, food refusal, other feeding refusal behaviours as well as dysphagia [25]. Up to one third of young children with Atopic Dermatitis (AD) may present with a FA, particularly those with moderate to severe eczema unresponsive to topical treatment [26,27]. Adults have less well documented non-IgE-mediated food allergies, but dysphagia and oesophageal food impaction are particularly associated with adult-onset EoE [28].

#### Presenting symptoms: other symptoms caused by food

Sometimes symptoms of an allergic disorder can resemble those due to non-immune mediated adverse reactions to foods, thus complicating the diagnostic process. For example, dietary histamine has been reported to provoke rhino-conjunctivitis, flushing, pruritus, urticaria, asthma, hypotension and abdominal cramping [29]. The food additive sodium metabisulphite has been linked to symptoms of rhinitis, nasal blockage, wheeze and abdominal pain [30]. Abdominal symptoms are a notable feature of Irritable Bowel Syndrome (IBS), a diagnosis which is positively associated with pain in the lower abdomen, pain relieved by bowel movements, frequent pain and abdominal bloating [31].

#### Onset of symptoms

It is important to establish the temporal relationship between eating a food and the onset of symptoms. It is generally agreed that the sooner the symptoms occur after eating, the greater the likelihood that an IgE-mediated mechanism is provoking the reaction [1,12]. Non-IgE-mediated FA symptoms are usually delayed; children with FPIES typically experience severe vomiting 1–4 hours after ingestion of the suspect food [32]. However occasionally symptom speed of onset does not provide such clear signposting; co-factors such as exercise can mislead if the symptoms only occur during exercise, but the food involved could have been eaten up to 2 hours earlier [33]. Another example is delayed anaphylaxis to red meat occurs in relation to IgE antibodies to the oligosaccharide galactose-alpha-1,3-galactose (alpha-gal) generated by tick bites [34]. Conversely, some non-immune-mediated adverse food reactions may manifest soon after eating, i.e. gastrointestinal symptoms triggered by lactose intolerance [35], or wheeze provoked by the food additive sodium metabisulphite [30].

Other important factors relating to symptoms include ascertainment of the quantity of food required to trigger symptoms, frequency and reproducibility of the reactions and the interval since the last reaction [1,11-13]. The age of onset of symptoms, feeding/diet history, history of any food elimination and any previous therapeutic interventions are also significant.

#### Atopic history

For children, in addition to a physical examination focusing on growth and development, any allergy related co-morbidities and family history of atopy should be ascertained [1,11]. The likelihood of food allergy is enhanced in children with moderate to severe eczema [26]. For adults, a physical examination is also helpful, but it is also vital to establish atopic status due to the strong association between atopic disease and the manifestation of food allergy in adults [36]. Allergic rhinitis is increasingly prevalent in food allergic adults, reportedly affecting 41% of UK adults suffering from adverse reactions to foods in 2009, compared to 25% in 1991 [37,38]. Asthmatics with food allergy are also more likely to have severe and uncontrolled asthma [39] and suffer from fatal or near fatal food anaphylaxis [22].

#### Known aeroallergen sensitisations

There is an increasing level of aeroallergen sensitisation in all age groups, which persists into old age [40,41]. Thus establishing sensitisation to pollens, mites and animal dander is vital when interpreting the history, especially as geographical variation in aeroallergen sensitisation across Europe affects prevalence and foods involved [42]. Positive tests to pollen [15], latex [43] or house dust mite [44], can be linked to homologous reactions to fruits, vegetables, nuts or shellfish. More unusual cross-reactions include sensitisation to bird feathers inducing symptoms to the egg yolk allergen alpha livetin [45], individuals sensitised to galactose-alpha-1,3-galactose (alpha-gal), reacting to beef and pork [34], and reactions to pork associated with sensitisation to cats [46]. The sensitising agent might be a food-related parasite such as cereal mites [47] or the *Anisakis* nematode worm [48].

#### Extrinsic (co) factors and routes of exposure

In adults, exercise, alcohol, stress, aspirin or non-steroidal anti-inflammatory drugs (NSAIDs) can enhance or precipitate an allergic reaction to food [49]. Food-dependant exercise-induced anaphylaxis (FDEIA) is characterised by a lack of reaction to the trigger food unless it is consumed in close proximity to taking exercise [50]. Wheat, crustaceans, tomatoes, celery, strawberries, cheese have all been implicated [33,49-53]. Alcohol can also act as a co-factor, but symptoms to alcohol could also be due to an allergy to grapes or barley [54,55], sensitivity to vasoactive amines or sodium metabisulphite [29,30], or caused by a congenital deficiency of the enzyme alcohol dehydrogenase [56]. Food additives have also been shown to act as co-factors in cases of anaphylaxis [53]. Co-factors may be less relevant in children [57], but exposure to allergens other than via the oral route require consideration in both children and adults; these include inhalation of aerosolized allergens, transfer of allergens during cooking or by skin contact [58-60].

### Food & symptoms

#### Food triggers – IgE-mediated FA

Although there are a large range of foods which can be implicated in IgE-mediated FA, only a limited number regularly provoke reactions in most people. Most reactions in children are due to milk, egg and peanut [2], with milk and egg being the most common causes of anaphylaxis [61]. Milk, egg, wheat and soy allergy usually remit in late childhood [62-65], thus a primary allergy to these foods is rare in adults. Wheat allergy in adults is generally associated with FDEIA [52] and adults reporting reactions to soy usually develop symptoms due to homology between soy protein and birch pollen allergens [66]. Peanut and tree nut allergy in adults is usually a persistence of childhood allergy since only 8-10% of cases are newly diagnosed in adolescence or adulthood [67-69]. Up to 30% of adults with a peanut allergy have cross-reactivity to other legumes such as lupine [70]. Lupine allergy also presents in childhood, but is far less prevalent than peanut allergy in the general population, affecting less than 0.3% of patients with reported reactions to foods [71]. Seeds such as sesame and mustard are also a significant cause of IgE-mediated reactions in certain populations [72,73]. Those with a primary allergy to one type of nut, seed or legume can react to other similar foods due to co-sensitisation to homologous allergens [74-79].

Fish and shellfish trigger both paediatric and adult IgE-mediated FA [80,81], often provoking severe reactions [22,82]. Pan allergens in vertebrate fish account for strong interspecies cross-reactivity; thus sensitization to more than one species is common in fish-allergic individuals [80]. The same is true for crustaceans and molluscs, although sensitisation to minor seafood allergens can limit clinical reactivity solely to the seafood which provoked the index reaction [83]. The lack of homology between the pan allergens in vertebrate fish and shellfish usually means fish-allergic individuals can tolerate shellfish and vice versa [80].

Fruits and, to a lesser extent, vegetables, are often the most frequently reported foods to provoke new onset reactions in adults [5,17,18,37]. These reactions are usually due to oral allergy syndrome or pollen-food syndrome (PFS), a common manifestation of cross-reactive plant FA in adults, which can affect 50% or more pollen-sensitized individuals [15,17]. Such reactions are also reported in older children and teenagers, although only limited epidemiological data supports this [84]. Reactions, usually to more than one food, typically occur to tree nuts, apples, peaches and cherries, although peanuts and soy may also be involved [15,17,85-87]. If PFS is suspected then any history of seasonal hay fever should be linked to symptoms to fruits and vegetables, and symptoms may be worse or only occur during the pollen season [61]. Homologous allergens in natural rubber latex (*Hevea brasiliensis*) can also cross-react with foods, in particular kiwi fruit, avocado, chestnuts and bananas [44]. Lipid Transfer Protein (LTP) allergy should also be considered if plant foods provoke severe reactions [88]. LTP allergy, usually associated with sensitisation to the peach LTP allergen Pru p 3 [88,89], is highly prevalent in southern Europe [90], and associated with presence of co-factors such as alcohol and exercise [19,49].

Whilst children might have a reaction to a food soon after the first exposure, older children and adults may suddenly develop symptoms to foods consumed for many years. However, foods habitually eaten are mostly considered as safe and should not be tested. If the reactions are to multiple foods and PFS is not suspected, then a ‘hidden’ allergen used as an ingredient in composite dishes may be implicated, such as legumes, seeds, celery and natural food colourings.

#### Food triggers – non-IgE-mediated FA

FPIES typically occurs in response to milk or soy proteins in infant formula, and more rarely to food proteins in breast milk [91,92]. The common provoking allergens in FPIES are milk, rice and soy, but reactions have also been reported to oats, fish, egg and some fruit and vegetables [93]. Although eosinophilic gastrointestinal disorders are seen in both adults and paediatrics, offending foods may differ [94]. In children, milk, egg, wheat and soy have been found to be most relevant, although in some cases fish, nuts and peanuts also play a role, although meat proteins (including, beef, lamb, chicken and pork), carrots, potato, maize and peanuts may also be causative foods [95]. Wheat and milk appear to be major allergens in adults although other triggers could include corn, rice and legumes [96].

#### Food triggers – non-immune mediated adverse food reactions

Milk is most frequently linked to non-immune mediated conditions in adults including lactose intolerance [35], IBS [97], and some respiratory conditions [98]. In the absence of IgE-sensitisation to wheat, coeliac disease [99] should be excluded prior to considering differential diagnoses such as non-coeliac gluten sensitivity [100] and IBS [101]. There is some evidence that foods containing high levels of oligo-, di-, mono-saccharides and polyols carbohydrates (FODMAPS™) could provoke some or all of the gut symptoms experienced by IBS sufferers [102]. Oligosaccharides (wheat, rye, onions, garlic, artichokes and legumes) probably affect the majority of IBS sufferers, 45% of whom will also be intolerant to foods high in fructose (honey, apples, pears, watermelon and mango) [103]. Reported reactions to multiple foods in the absence of IgE-sensitisation can indicate a non-immune mediated reaction. Potential mediators may include food additives (azo dyes, benzoates, sulphites and mono-sodium glutamate) [30,104], or naturally-occurring substances such as vasoactive or biogenic amines (fish, pork and fermented or aged meat products, strong cheeses, red wine, spinach, aubergine and yeast extract) [29], or salicylates (coffee, dried herbs and spices, cherries, strawberries and certain apple varieties) [105] although robust evidence on the prevalence of these types of reactions is lacking.

### Foods eaten and avoided

All foods and food products which can be consumed without any symptoms should be noted, especially common allergenic foods, or ‘hidden’ food allergens such as mustard, celery, soy and lupine. Emerging evidence indicates that some children can tolerate foods containing well-cooked milk and egg, but will still react to raw/less well cooked forms [106,107]. Similarly those with PFS may be able to tolerate cooked fruits but not raw [15,17]. Reported avoidance of the trigger food may not be complete exclusion [108] due to lack of awareness of foods that contain small amounts of allergen. A dietetic consultation will establish true abstinence and information from food labels, recipes or food diaries may also be useful [109].

#### Nutritional issues

Nutritional impairment is a tangible risk in those with suspected FA; the elimination of food allergens often entails the exclusion of foods that contribute essential nutrients for growth and development (Table 2) [9,10]. Poor growth and stunting has been shown to occur in children with IgE-mediated FA who are on exclusion diets [10,110], with the number of foods excluded linked to a low weight for age and height for age in such children [10]. Feeding difficulties may also occur as clinical features of non-IgE mediated FA. Adults generally are not in danger of developing major nutritional deficiencies although individuals avoiding multiple foods can be at risk [111]. Therefore both tools contain a nutritional assessment section which, on completion, should indicate whether onward referral for specialist nutritional intervention is required. A dietitian can ascertain and interpret the diet history to ensure normal growth and development, employing a variety of measures to determine dietary intake [112,113]. The nutritional analysis can signpost the requirement for nutritional supplements and give expert advice to ensure a healthy, regular and varied diet is taken as this has been shown to be of importance in the development of allergy [114,115] It is important that the user take into account the important role of under-nutrition in paediatric and adult food allergy since poor nutritional status may aggravate symptoms and a healthy diet could be important in the prevention of allergic disease [10,116,117].

**Table 2:** **Main food allergens and their nutrient content [** 118 **]**

**Allergen**	**Nutrients involved**
**Milk**	Protein, Carbohydrate, Fats, Vitamin A and vitamin D, riboflavin, pantothenic acid, vitamin B12, calcium, magnesium, phosphate
**Egg**	Protein, Riboflavin, biotin, vitamin A, vitamin B12, vitamin D, vitamin E, pantothenic acid, selenium, iodine, folate
**Fish**	All fish: Protein, iodine.
	Fish bones: calcium, phosphorus, fluoride.
	Fatty fish: Protein, fat, vitamins A and D, omega-3 fatty acids
**Shellfish**	Similar nutrients to white fish.
	Crab and mussels: Protein and good sources of omega 3
	Selenium, zinc, iodine and copper
**Molluscs**	Varying amounts of protein (scallop), calcium (clam), zinc (oysters) and iron (clam)
**Wheat**	Carbohydrate, Protein Fibre, thiamine, riboflavin, niacin, calcium, iron, folate if fortified
**Peanut**	Protein, fats, Vitamin E, niacin, magnesium
**Soya**	Protein, Thiamine, riboflavin, pyridoxine, folate, calcium, phosphorus, magnesium, iron, zinc, fibre
**Lupin**	Negligible nutritional value when consumed as a condiment or taken in very small amounts
**Tree nuts**	Depends on type of nut, but similar to peanuts + omega 3 and 6 fatty acids
**Sesame seed**	Protein, fats, Vitamin E, calcium, potassium, phosphorus, vitamin B and iron and omega 6 fatty acids
**Mustard**	Negligible nutritional value when consumed as a condiment or taken in very small amounts
**Celery/celeriac**	Fibre

### Amber stage - interpretation of history

This stage involves linking the answers to the questions from the ‘red’ section and includes interpretation of symptoms, medical history, trigger foods and nutritional issues. Typical symptoms and foods associated with IgE mediated FA are listed in the left hand column of this section, and adapted to differences in age-related onset. The right-hand column lists foods and symptoms most associated with adverse food reactions that are not immune-mediated. In general, paediatric patients are far more likely to suffer from non-IgE mediated conditions than non-immune mediated conditions [24]. Thus the paediatric tool also contains a middle column listing foods and symptoms most associated with paediatric non-IgE mediated FA. However, this middle column is not present on the adult tool since these conditions are often most prevalent in childhood [24], the exception being EoE, which does exist in adults, with the adult form usually being diagnosed between the ages of 30–50 years [119].

This section also contains a guide to the foods most likely to be causative of FA at different ages and to support the interpretation of reported food triggers. There is information on cross-reactivity, a PFS diagnostic algorithm [15] and lists of potential allergens in composite meals. Collectively, the information in the amber section enables the user to decide whether the patient has an IgE- or a non IgE-mediated FA, a non-immune mediated adverse food reaction or a differential diagnosis not related to allergy, or combined symptoms.

### Green stage - tests and diagnosis

This section contains a test algorithm and care plan to summarise the outcome. Once the green stage is completed, the user should have sufficient information to undertake further tests, or make an onward referral to specialist services as indicated.

## Conclusion

The EAACI Food Allergy and Anaphylaxis Guidelines [1] emphasise the importance of taking a careful allergy-focussed history, and asking structured questions. These age-appropriate diet history tools, developed by a multi-professional international group of experts, bring together relevant factors, important in the aetiology of FA. The authors acknowledge that all of the evidence submitted in support of the tools has not been systematically graded, however, this is the first time that all of the relevant questions in an allergy focussed diet history have been agreed, documented, referenced and linked in a systematic way to provide standardised practical tools. The paediatric and adult diet history tools can be adapted to country specific dietary norms, whilst ensuring a standardised approach and screening for nutritional status. The information gained is vital in the formulation of a provisional diagnosis.

These tools can be used by anyone who needs guidance in taking a diet and clinical history, being particularly useful for those who not working in a specialist allergy setting. An accurate history determines the need for allergy testing. Once a diagnosis is made, patients can be managed appropriately or be referred onwards to an allergy specialist physician and/or dietitian as required. Newly diagnosed food allergy is a growing burden for all ages, against a backdrop of complex dietary intake patterns, multiple co-factors, social complications and diverse presenting features. These tools provide a scaffold of questions on which the diagnosis of FA can be built.

## Additional files

Additional file 1:
**Allergy-Focussed Diet History Paediatric version.**
Additional file 2:
**Allergy-Focussed Diet History Adult version.**
