# Supplementary material for: The development of a standardised diet history tool to support the diagnosis of food allergy
Source: Clin Transl Allergy. 2015 Feb 19;5:7. doi: 10.1186/s13601-015-0050-2 (PMC4349299; doi:10.1186/s13601-015-0050-2)
Supplement: Additional file 1: — Allergy-Focussed Diet History Paediatric version. [file 13601_2015_50_MOESM1_ESM.pdf]

Allergy-Focussed Diet History

Paediatric version

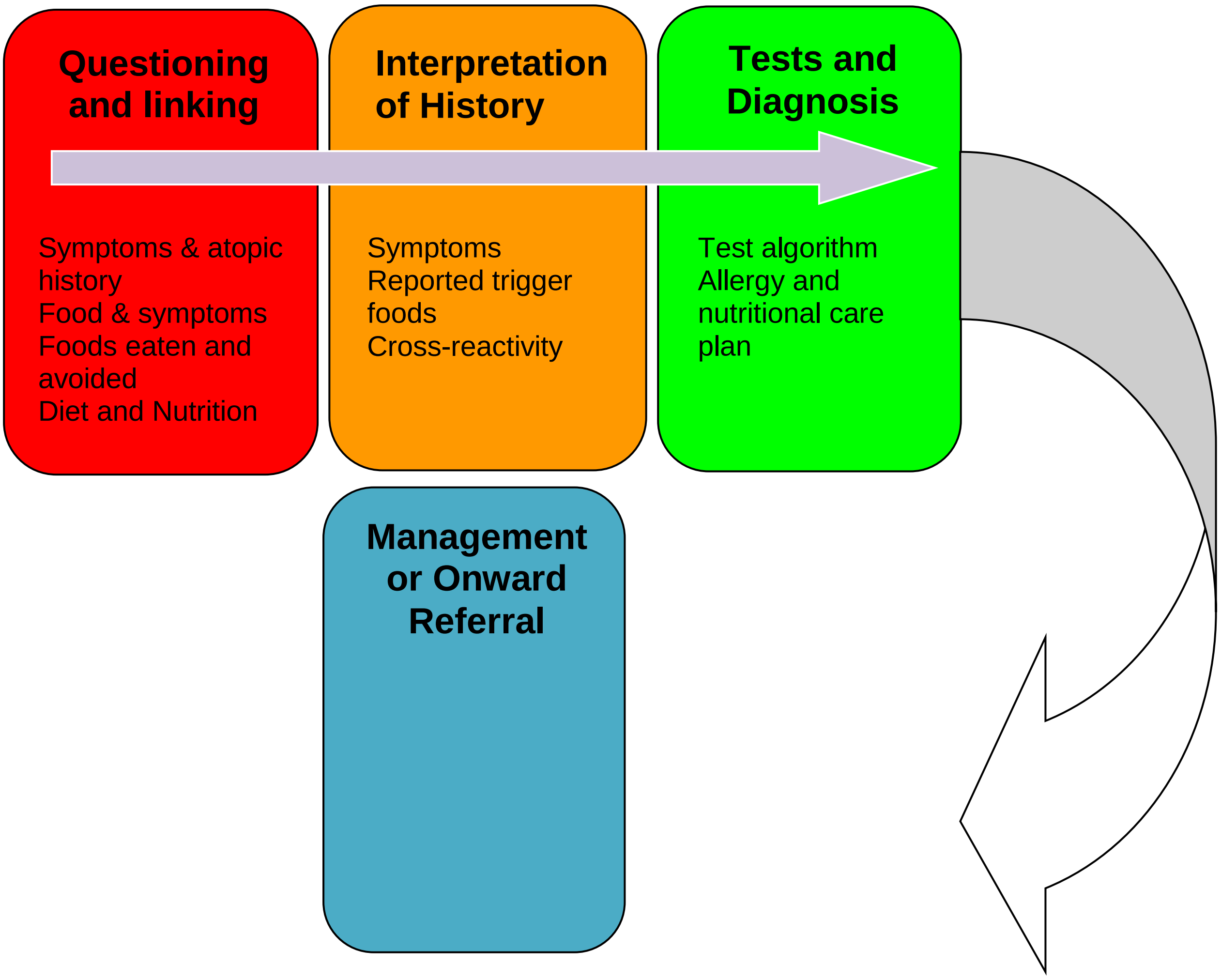

Contents

	Page
**Introduction**	**3**
How to use this tool	3
Defining the terms	3
Abbreviations	3
**Questioning and linking**	**4**
Symptom and Atopic history	4
Food and Symptoms	7
Foods eaten and avoided	10
Diet and Nutrition	11
**Interpretation of History**	**12**
Interpretation of symptoms	12
Interpretation of reported trigger foods	13
Linking foods eaten to specific allergens	15
Linking age to foods	18
Linking cross-reactivity between botanically related foods and foods and aeroallergens	19
**Tests and Diagnosis**	**20**
Pollen-food syndrome (Oral allergy syndrome) Algorithm	20
Allergy and nutritional care plan	21
**References**	**22**
**Appendices**	**25**
A. Task Force Membership	25

Introduction

The European Academy of Allergy and Clinical Immunology (EAACI) Guidelines on food allergy (FA)[1] suggest that the allergy-focussed history is fundamental to the establishment of the likelihood of a diagnosis, and the mechanisms and food triggers involved. In recognition of this, the Allied Health Interest group of the European Academy of Allergy and Clinical Immunology (EAACI) established a Task force (see Appendix A) to develop allergy-focussed diet history tools to facilitate a systematic standard approach to the diagnosis of infants and children with suspected food allergy, and support best practice and the development of pathways of care.

How to use this tool

This tool has been designed to support food allergy diagnostic pathways and determine when onward referral and/or specialist dietetic or nutritional intervention is required. Infants and young children presenting with suspected food allergy are especially vulnerable to growth and nutritional deficiencies so require optimal diagnosis and management [1,2]. This tool is not profession-specific, and can be used in its entirety by those less skilled in allergy diagnosis, or as an aide-memoir for those who are working in the allergy specialist field. It can also be utilised as an educational tool when teaching health care practitioners about food allergy.

Defining the terms

Atopy	A personal and/or familial tendency, usually in childhood or adolescence, to become sensitised and produce IgE antibodies in response to ordinary exposure to allergens, usually proteins.
Food Allergy (FA)	An adverse reaction to food mediated by an immunological mechanism, involving specific IgE (IgE mediated), cell–mediated mechanisms (non IgE mediated) or both IgE and cell mediated mechanisms (mixed IgE and non IgE mediated).
.	
IgE-mediated FA	Immunoglobulin E mediated and is thought to manifest as a phenotypical expression of atopy, together with (or in the absence of) atopic eczema, allergic rhinitis and/or asthma.
Non-IgE mediated FA	Cell-mediated allergy and presents mainly with gastro-intestinal symptoms in reaction to the ingestion of a food allergen.

Abbreviations

FA	Food Allergy
FHS	Food Hypersensitivity
PFS	Pollen-food Syndrome
FPIES	Food Protein Induced Enterocolitis Syndrome

Symptom and Atopic history

*Indicates the potential need for onward referral for specialist allergy and/or dietary assessment

**1**	**Name**____<br><br>**Age** __________ **Male/Female**<br>**Height/length**______________**Weight**_____________
**2**	**Presenting symptoms**[1, 3,4] and pattern of appearance (Tick box and circle relevant symptoms)<br>☐ **Skin**: flushing/erythema, pruritus, urticarial*, angio-oedema*, eczema*<br>**Pattern** - Intermittent (on one occasion or weekly, monthly, annually) OR Continuous<br>☐ **Oro-pharyngeal –** pruritus, oedema (lips, tongue, pharynx), vocal changes (laryngeal oedema)*, throat closure*<br>**Pattern** - Intermittent (on one occasion or weekly, monthly, annually) OR Continuous<br>☐ **Gastro-intestinal**:, dysphagia, acute abdominal pain, colicky abdominal pain nausea, vomiting, diarrhoea/loose frequent stools, blood or mucous in the stool*, gastro-oesophageal reflux disease, peri-anal redness, back arching<br>**Pattern** - Intermittent (on one occasion or weekly, monthly, annually) OR Continuous<br>☐ **Upper and lower airway**: conjunctivitis, nasal itching, sneezing, rhinorrhoea with or without conjunctivitis, cough, chest tightness*, wheeze*, shortness of breath*, stridor*<br>**Pattern** - Intermittent (on one occasion or weekly, monthly, annually) OR Continuous<br>☐ **Cardiovascular*** – dizziness, hypotension, tachycardia, hypotonia (collapse)<br>**Pattern** - Intermittent (on one occasion or weekly, monthly, annually) OR Continuous<br>☐ **Anaphylaxis*** [5-7] – multi-system involvement e.g. skin symptoms plus respiratory or cardiovascular symptom, or two or more symptoms from different symptom categories<br>☐ **Other -** Pallor, tiredness, faltering growth*, malnutrition* or other condition:<br>____<br><br>**Pattern** - Intermittent (on one occasion or weekly, monthly, annually) OR Continuous
**3**	At what age did the symptoms first appear? _________________________<br><br>In what circumstances did symptoms first appear? (during or following a meal, location

	etc.)<br>____
**4**	Where do the reactions normally take place:<br><br>Home school* nursery/day-care* restaurant/takeaway* other location
**5**	Has any treatment/medication been given? – Yes No<br><br>If yes - what was it and did it help
**6**	Are any extrinsic factors involved(8,9) : Yes* No<br><br>If Yes – which ones:<br><br>infection medication (NSAIDS exercise fatigue hormonal alcohol
**7**	Current and/or previous concomitant conditions:<br><br>☐ Asthma(7) *<br>Age onset…………….<br><br>On-going problem: : Yes No<br><br>Medication …………………. Symptoms: mild/moderate/severe<br><br>☐ Allergic rhinitis (10)* (depending on severity)<br>Age onset……………….<br><br>On-going problem: Yes No<br><br>Seasonal - Spring/Summer/Autumn/ OR Perennial ______________<br><br>Medication ………………… Symptoms: mild/moderate/severe<br><br>☐ Eczema(11)* (depending on severity)<br>Age at onset………………………<br><br>On-going problem Yes No<br><br>Medication ……………………………… Symptoms: mild/moderate/severe<br><br>☐ Other co-morbidity * (depending on severity and type)

	Are these well controlled?
**8**	Parental/sibling history of atopy:<br><br>Asthma allergic rhinitis food allergy eczema
**9**	Does the child have disrupted sleep Yes No<br>(i.e. Excessive waking related to discomfort)<br><br>Does this continue: all night certain parts of the night
**10**	Do the parents suspect food allergy Yes No<br><br>If yes, which foods ____
**11**	Is the child known to be sensitised to or reports previous positive tests to;<br><br>☐ Aeroallergens (if yes please circle which ones)<br><br>Grass Trees Weeds Mites Cockroaches Moulds Animal dander<br><br>Other ____<br><br>☐ Foods (if yes please circle which ones)<br><br>Milk Egg Fish Shellfish Wheat Nuts Peanuts Seeds<br><br>Other____

For interpretation of symptoms: see page 12

Questioning and linking

Symptom & atopic history
Food & symptoms
Foods eaten and avoided
Diet and Nutrition

Food and Symptoms

*Indicates the potential need for onward referral for specialist allergy and/or dietary assessment

**1**	Feeding history: Breastfed formula feed N/A
**2**	If breast fed, review maternal diet: are there any foods being avoided or being consumed in excessive amounts
**3**	a) What type of infant formula or milk substitute is the child taking (tick as appropriate)<br><br>☐ Standard infant cow's milk formula/follow-on formula with/without prebiotics/probiotics<br>☐ Partially/Extensively hydrolysed casein/whey formula - type________________<br>☐ Partially/extensively hydrolysed rice formula - type ________________________<br>☐ Amino acid formula – type ____________________<br>☐ Infant soy formula – type __________________<br>☐ Non-fortified soy milk – type____________________<br>☐ Fortified soya milk – type_______________________<br>☐ Other milk: Fortified yes/no - type________________________________<br><br>b) How much formula is taken per 24 hours ___________________<br>*if consuming < 600 ml and below 1 year[12]
**4**	Have complementary foods been introduced in the diet of the child Yes/ No<br><br>If yes at what age _____________________ If no go to Q6

	**Food**	**Age**	**Format** – in what form was food given?	**Any problems** with weaning or with particular foods e.g. colic or reflux?
**5**	Fruits			
	Vegetables			
	Rice/corn			
	Meat/chicken			
	Fats/oils			
	Milk			
	Egg			

	Wheat			
	Cod or other white fish			
	Salmon or other oily fish			
	Shellfish			
	Soya			
	Peanuts			
	Tree nuts (specify which ones)			
	Seeds			
**6**	Is the child refusing food/to feed Yes No<br><br>If Yes Is this refusal associated with back arching or distress/crying? Yes No			
**7**	Is the child experiencing early satiety or consuming only small portions Yes No			
**8**	Have foods been eliminated previously<br><br>Yes No If Yes - was this helpful? Yes No			
**9**	Are symptoms related to a specific food? Yes / No / Possibly<br>If no complete Q10, if yes or possibly complete Q 11 on next page			
**10**	If no specific food identified, list the meals preceding the most recent reaction and two other reactions including the most severe (think of age-related foods and possible cross-reacting foods to inhalant allergens)			

Meal	Time to onset of symptoms (minutes)	Symptom Type

11	Are any foods identified which provoke symptoms? – (consider botanically related[a] or cross-reacting[b] allergenic foods)				
Food	Symptom type	Speed of onset[a]	Amount provoking reaction[b]	Raw or cooked	Symptoms every time food is eaten
(a) How many minutes or hours after eating did symptoms appear<br>(b) How much food provokes a reaction e.g. touching lips/inhalation* a mouthful* a few bites whole meal or snack several days of consuming the food					

For interpretation of Trigger foods, see page 13

For cross-reacting foods, see page 16

Questioning and linking

Symptom & atopic history
Food & symptoms
Foods eaten and avoided
Diet and Nutrition

Foods eaten and avoided

NB – please also list any foods the Mother is avoiding if breastfeeding

For interpretation of Trigger foods, see page 13

	Avoided	Eaten
**Milk - c**ow, sheep or goats milk, cheese, yoghurt, butter		
**Egg -** hen, duck, quail		
**Tree nuts -** Hazelnut, almond, walnut, Brazil nut, , pecan, cashew, pistachio, Macadamia nut		
**Peanuts and Legumes -** soy, chickpeas, peas, beans, lentils, lupin		
**Seeds** - sesame, sunflower, pumpkin, poppy, mustard, pine nuts		
**Fresh fruit, juice or smoothies** e.g. apple, kiwifruit, peach, strawberry, banana, mango, avocado		
**Fresh vegetables and vegetable juices** e.g. tomato or carrot		
**Herbs and spices** e.g. coriander, parsley, chilli, cumin, paprika		
**Cereals -** Wheat, rice, barley, oats, corn, buckwheat (not a cereal), rye, spelt, quinoa		
**Fish** (white or oily) e.g. cod, salmon, trout		
**Shellfish –** e.g. Prawns, mussels, squid		
**Meat , poultry and game**		
**Beverages -** Cordial/squash, fizzy drinks, alcohol		
**Not certain or multiple foods -** Compare the daily diet to that in Appendix C to ascertain which allergens might be involved		

D. Diet and Nutrition

NB – All children with a suspected food allergy should be referred for an assessment of nutritional adequacy and imbalance

**1**	Is the weight for height and height for age ≤ 2 z-score(12)	Yes*	No
**2**	Has there been:<br>Recent growth faltering: i.e. wasting (low weight for height) or stunting (low height for age) or downward crossing of ≥ 2 centile in weight or length<br>Yes* No<br><br>OR Static weight(13) Yes* No		
**3**	Does the child have age-appropriate progression in oral motor skills? see page 20	Yes	No
**4**	Are multiple foods being avoided?	Yes*	No
**5**	Has one or more foods been excluded for 6 months or longer?<br>Yes* No		
**6**	Is the patient vegetarian or vegan?	Yes*	No
**7**	Any fortified foods or nutritional supplements being taken?<br>(e.g. foods fortified with energy, calcium or other nutrients)<br>NB consider national recommendations regarding vitamin D supplementation	Yes	*No
**8**	Any religious or cultural factors that can affect food intake?	Yes*	No
**9**	Does the child have a long standing chronic condition that further impairs dietary intake?<br><br>Yes* No		

***At high risk of nutritional inadequacy**

Interpretation of Symptoms

		Likely IgE-mediated FA[1, 3,4]	Likely non-IgE-mediated FA	Differential Diagnosis
SYMPTOM TYPE	**Skin**	Pruritis -extremities and groin<br>Urticarial rash of less than 48 hours duration<br>Acute rapid-onset angio-oedema after eating	Atopic dermatitis	Non-specific pruritis<br>Delayed onset urticarial rash of more than 48 hours duration<br>Hereditary angio-oedema<br>Viral urticarial, chronic urticarial, atopic eczema,<br>Angio-oedema several hours after eating
	**Gastro-intestinal**	Oro-pharyngeal pruritis (OAS), oedema, tingling, paraesthesia and dysesthesia<br>Severe, acute intermittent vomiting and/or diarrhoea within 30 minutes of eating	Chronic constipation or soft stool constipation associated with straining<br>Diarrhoea with/without mucus and/or blood and/or vomiting<br>Repeated vomiting<br>Abdominal distension/bloating<br>Feeding aversion<br>Collapse/clinical picture similar to sepsis (FPIES)<br>Back-arching following feeds	Chronic constipation or diarrhoea<br>Bloating<br>Acute I GI pain<br>Gastro intestinal infection
	**Respiratory**	Rhinitis, conjunctivitis, dyspnoea, wheeze, stridor, acute-onset difficulty in breathing		Respiratory tract infection<br>Asthma exacerbation<br>Inhalation of a corpus alienum
	**Circulatory**	Tachycardia or Hypotension	Hypotension (in FPIES)	Cardio vascular problems
**SPEED OF ONSET**		IMMEDIATE<br>Up to 2 hours after eating	INTERMEDIATE<br>More than 2 hours after eating or up to 2 days with continued exposure	DELAYED<br>More than 2 days after eating or not linked to food
**SEVERITY**		Anaphylaxis, difficulty in breathing	Hypovolemic shock (FPIES)	
**REPRODUCIBILITY**		Symptoms every time same food eaten		No discernible pattern

Interpretation of History

Symptoms
Reported trigger foods
Age
Cross-reactivity

Interpretation of Reported Trigger Foods

Reported Food	Likely IgE-mediated FA	Likely non-IgE-mediated FA	Likely non-allergic FHS or Differential Diagnosis
**Milk**	Milk, cheese, yoghurt, fromage frais, ice cream, milk in foods, butter	Milk, cheese, yoghurt, fromage frais, ice cream, milk in foods	Milk and soft cheese (Lactose intolerance[14])
**Egg**	Egg, baked egg, runny egg	Egg, baked egg, runny egg	
**Tree nuts** NB in older children, to check cross-reactivity, apply PFS Algorithm (Appendix C)[6]	Hazelnut, almond, brazil nut, walnut, pecan, cashew, pistachio, macadamia)		
**Peanuts and other Legumes** NB in older children, to check cross-reactivity, apply PFS Diagnostic Algorithm (Appendix C)	Peanuts		
	Soy (immediate-onset symptoms)	Soy	
	Lupin		
	Chickpeas, lentils, etc.		
**Seeds**	Sesame, sunflower, pumpkin, mustard, pine nuts, poppy seeds		
**Fresh fruit** NB in older children, to check cross-reactivity, apply PFS Algorithm (Appendix C)	Any fresh raw fruit especially apples, strawberries, plums, cherries, peaches, pears, kiwi	Strawberries	Pineapple or oranges – non-specific oral rash carbohydrate malabsorption (i.e. fructose) secondary due to GI inflammation[15]
**Fresh vegetables** NB in older children, to check cross-reactivity, apply PFS Algorithm (Appendix C)	Any fresh raw vegetable, especially carrots, peppers, celery, tomatoes, avocado		Tomatoes – non-specific oral rash
**Herbs and spices**	Fresh herbs, especially		

	coriander and parsley,<br>mustard, chilli, paprika,<br>celery salt, curry powder		
**Cereals**	Wheat<br>Rice<br>Barley<br>Corn – e.g. polenta,<br>cornflakes, tortilla	Wheat<br>Barley<br>Rice<br>Corn<br>Oats	Gluten intolerance
**Seafood**	Finned Fish (cod, herring,<br>mackerel, sea bass, plaice<br>etc.)<br>Crustaceans (prawns, crab,<br>lobster, gamba, langoustine<br>Molluscs (mussels, clams,<br>oysters		All seafood can cause<br>toxic reactions – usually<br>late onset nausea and<br>vomiting
**Meat**	Beef, lamb, pork – delayed<br>onset severe anaphylaxis<br>Chicken, processed ham<br>and chicken (contain milk)<br>Sausages (soy)		
**Meat substitutes**	Mycoprotein (egg)<br>Vegetarian sausages,<br>burgers or patties (soy,<br>other legumes or nuts)		
**Beverages**	Fruit juices containing milk<br>solids		Lime cordial, bottled<br>lemon/lime juice
**Composite meals<br>or snacks**	Meals containing nuts,<br>seeds and seafood (UK -<br>Indian curry, Thai,<br>Malaysian, Chinese),<br>Hummus, Pesto, chips (if<br>allergic to fish), chicken<br>nuggets, fried chicken	Pizza, Pasta, Mexican, Italian, burgers<br>Soup gravy and sauces	

Interpretation of History

Symptoms
Reported trigger foods
Cross-reactivity

Linking foods eaten to specific allergens -

Daily food pattern and possible allergens (adapted from Food Hypersensitivity, Skypala and Venter[16])*

	Milk	Egg	Wheat	Soy/ Legumes	Peanut/ Tree nut	Other allergens
**Breakfast**						
Breakfast cereal	✓		✓		✓	Barley
Bread	✓		✓	✓	✓	Barley
Butter/Margarine	✓					
Chocolate spread					✓	
Muffin, Pastry or croissant	✓	✓	✓	✓	✓	
Ham/Bacon	✓					
Sausage		✓	✓	✓		
**Snack meal**						
Soup			✓	✓		Celery, mustard
Bread roll	✓	✓	✓	✓	✓	Seeds
Sandwich	✓	✓	✓	✓		
Salad dressing	✓	✓	✓	✓		
Vinegar/ketchup			✓			Barley
Biscuits	✓	✓	✓	✓	✓	
Cake	✓	✓	✓	✓	✓	Cochineal
Custard	✓					Annato
Hummus				✓	✓	Sesame
Pesto	✓				✓	
Crisps	✓					
**Main meal**						
Baby jars	✓	✓	✓	✓		Fish, celery
Baby cereals	✓		✓			
Baby pouches						Celery
Fish in batter		✓	✓			
Sausages		✓	✓	✓		
Thai/Malaysian			✓		✓	Sesame, seafood, buckwheat
Chinese		✓	✓	✓	✓	seafood
Curry	✓		✓	✓	✓	Coriander cumin, turmeric, fenugreek, celery, mustard
Pizza	✓	✓	✓	✓		Celery, mustard
Fruit tart/fritters			✓	✓		Lupin
Yoghurt/ice cream	✓	✓			✓	
**Other**						
Gluten-free foods						Lupin

***This table should be adapted for each country**

Interpretation of History

Symptoms
Reported trigger foods
Cross-reactivity

Linking foods eaten to specific allergens -

Foods likely to contain milk, egg, cereals, peanuts or tree nuts

	Milk	Egg	Wheat & Barley	Peanuts & Tree nuts
**Bread and breakfast cereals**	Breakfast cereals, some breads	French toast	Bread (sourdough, nan, soda), breakfast cereals, pancakes, muffins, crackers	Breakfast cereals, peshawari nan bread, almond croissants
**Meat, fish, egg, cheese, vegetarian dishes**	Packet sliced cold meats, lasagne, sausages, foods in batter, sandwiches, quiche	Sausages, fish fingers, foods in batter/breadcrumbs	Sausages, foods in batter/breadcrumbs, pies, meat puddings, sandwiches	
**Pasta, rice, potato vegetables,**	Pasta in cheese or cream sauce, baked beans, soups,	Yorkshire pudding, egg fried rice, egg pasta, meat substitute. bouillon	Potato cakes, couscous, pasta, spelt, baked beans	Vegetarian dishes
**Desserts, Cakes and biscuits**	Yoghurt, milk pudding, cheesecake, pancakes, custard, ice cream, mousse, Cakes, biscuits, muffins	Pancakes, meringue, soufflé, sponge, trifle, crème brulee, egg custard, pastry cream, mousse, ice cream, cakes, pastries brushed with raw egg, marzipan, royal icing,	Semolina, cheesecake, tarts, sponge, crumble, pancakes, biscuits, cakes, pastries,	Pastries, nut cookies, Ice cream toppings, Bakewell tart, brownies, fruit cake, nut cookies, marzipan, hazelnut paste, halva, baklava, macaroons
**Restaurant and take away food**	Pizza, curry containing cream (e.g. korma)			Curry especially Korma sauce, Thai and Chinese food, Satay sauce, Szechwan sauce
**Sweets and snacks**	Toffee, fudge. Caramel, chocolate, crisps, flavoured snacks	Filled chocolates, cream/fondant fillings		Peanut/nut brittle, Marron glace, sugared almonds, praline, chocolates, nougat
**Condiments and spreads**	Salad dressing, butter, margarine	Salad dressing, mayonnaise, salad cream, Hollandaise sauce, lemon curd	Sauces, gravy	Peanut butter, Chocolate hazelnut spread, cold pressed walnut, almond and hazelnut oil peanut/groundnut oil
**Drinks**	Infant formula, milk, pineapple & coconut juice, latte, coffee and tea whiteners		Malt drinks	

***This table should be adapted for each country**

Interpretation of History

Symptoms
Reported trigger foods
Cross-reactivity

Linking foods eaten to specific allergens -

Foods likely to contain soy, lupin, sesame, mustard and sulphites

	Soy & Lupin	Sesame	Mustard	Celery
**Bread, crackers, breakfast cereals**	Bread, crackers, gluten-free products	Bread sticks, bagels, crackers, crispbread, rice cakes		
**Meat, fish, egg, cheese, vegetarian dishes**	Tofu, tempeh, sausages, beef burgers baby food, battered food	Burger buns, falafel, samosa	Cheese sauce, Welsh Rarebit, ready meals, stews, casseroles, hot dogs	Ready meals, casseroles, stews
**Pasta, rice, potato vegetables,**	Endamame beans, noodles	Noodles	Pickled vegetables	Vegetable puree
**Desserts, cakes and biscuits**	Biscuits, pastries	Baklava, halva, sesame snaps, pastries,		
**Restaurant and take away food**	Pizza	Chinese and Thai food	Curry, Pizza, Mexican	Curry, pizza
**Sweets and snacks**	Cheese dips	Hummus,		
**Condiments and spreads**	Soy sauce, stock cubes, sauces, mayonnaise, salad dressing	Salad dressing, soups, sauces, dips	Salad dressing, barbeque sauce, soups, ketchup, Mayonnaise, marinades, piccalilli, chutney, curry powder	Curry powder, spice mixes, rubs, sauces, marinades, soups, salad dressing, Yeast extract, gravy, stock cubes, ketchup, Barbeque sauce, chutneys, pickles
**Drinks**	Soy milk, miso soup, body-building protein shakes			Vegetable juice, tomato juice

***This table should be adapted for each country**

Interpretation of History

Symptoms
Reported trigger foods
Age
Cross-reactivity

Linking age to foods suspected

Age	Foods most frequently associated with IgE-mediated FHS[1,3,4]
**Infants (0-5 years)**	Cow's milk, egg, peanut, soy, nuts
**Young children (5-10 years)**	Cow's milk, egg, peanut, tree nuts, wheat, soy (fish, kiwifruit, sesame)
**Older children 10-16 years**	Peanuts, tree nuts, seeds, fish, fruits, vegetables

***This table may need to be adapted for each country due to differences in diet and aeroallergen sensitisation patterns**

Interpretation of History

Symptoms
Reported trigger foods
Cross-reactivity

Linking cross-reactivity between foods and aeroallergens [17-36]

Primary sensitisation or allergy to:	Potential co-sensitisation or cross-reactions to:
**Pollens**	
Silver Birch	apple, pear, cherry, peach, nectarine, apricot, plum, damson, greengage, strawberry, kiwifruit, hazelnut, walnut,, almond, Brazil nut, celery, carrot, potato, soy, fig, bean sprouts, mangetout
Plane tree	hazelnut, peach, apple, melon, kiwi, peanuts, maize, chickpea, lettuce, green beans
Grass	melon, watermelon, orange, tomato, aubergine, sweet pepper, chilli or cayenne pepper, potato, peanut, Swiss chard
Mugwort	celery, celeriac, carrot, parsnip, dill, parsley, coriander, cumin, fennel, aniseed, caraway, angelica, chervil, sunflower seed, honey
Ragweed	watermelon and other melon, banana, courgette, cucumber, courgette, marrow, squash, pumpkin,
Latex	Avocado pear, chestnut, peach, banana, passion fruit, kiwi fruit, papaya, mango, tomato, pepper, potato, celery
**Foods**	
Peanut	Walnut, pistachio nut, cashew nut, Hazelnut, almond, Brazil nut, sesame seed, other legumes (see below)
Pistachio or cashew nut	Pistachio or cashew nut, peanut, hazelnut
Walnut or pecan nut	Walnut or pecan nut, hazelnut, cashew nut, peanut, sesame
Hazelnut	Cashew, Brazil nut, pistachio, almond, peanut, walnut
Sesame	Sunflower seeds, peanuts, walnuts, hazelnut, Brazil nut, almonds
Peaches	Apples, hazelnuts, peanuts, walnuts, orange, cabbage, lettuce, mustard, corn, barley

Legumes	Peanuts, Soy, Pigeon Pea, Goa Bean, Runner Bean, French Bean, Haricot Bean, Butter Bean, Lima Bean, Chick Pea, Mung Bean, Fava Bean, Peas, Lentils, Tamarind, Guar Gum, Fenugreek, Liquorice, Gum Arabic, Tragacanth

Interpretation of History

Symptoms
Reported trigger foods
Cross-reactivity

Age-appropriate progression of oral-motor skills [37-40]

Age (months)	Oral-motor skills
**0-6 months**	Sucking and swallowing from the breast/bottle. If weaning occurs > 17 weeks < 6 months, able to remove pureed foods off a spoon.
**6-10 months**	Able to remove foods of a spoon. Texture initially puree with soft lumps* and progresses within 6-10 months to bigger lumps and finger foods. Able to chew soft foods and has good lateral tongue movement. Able to hold spoon and starts to attempt to assist in feeding. Drinking from a feeder beaker/cup without help.
**>10 months**	Eats pieces that require advanced chewing, able to finger feed starts to be able to spoon feed. Able to drink independently from a cup.

***It is critical to have lumpy textures at 10 months of age**

©Pollen-Food Syndrome (Oral Allergy Syndrome) Algorithm(17)

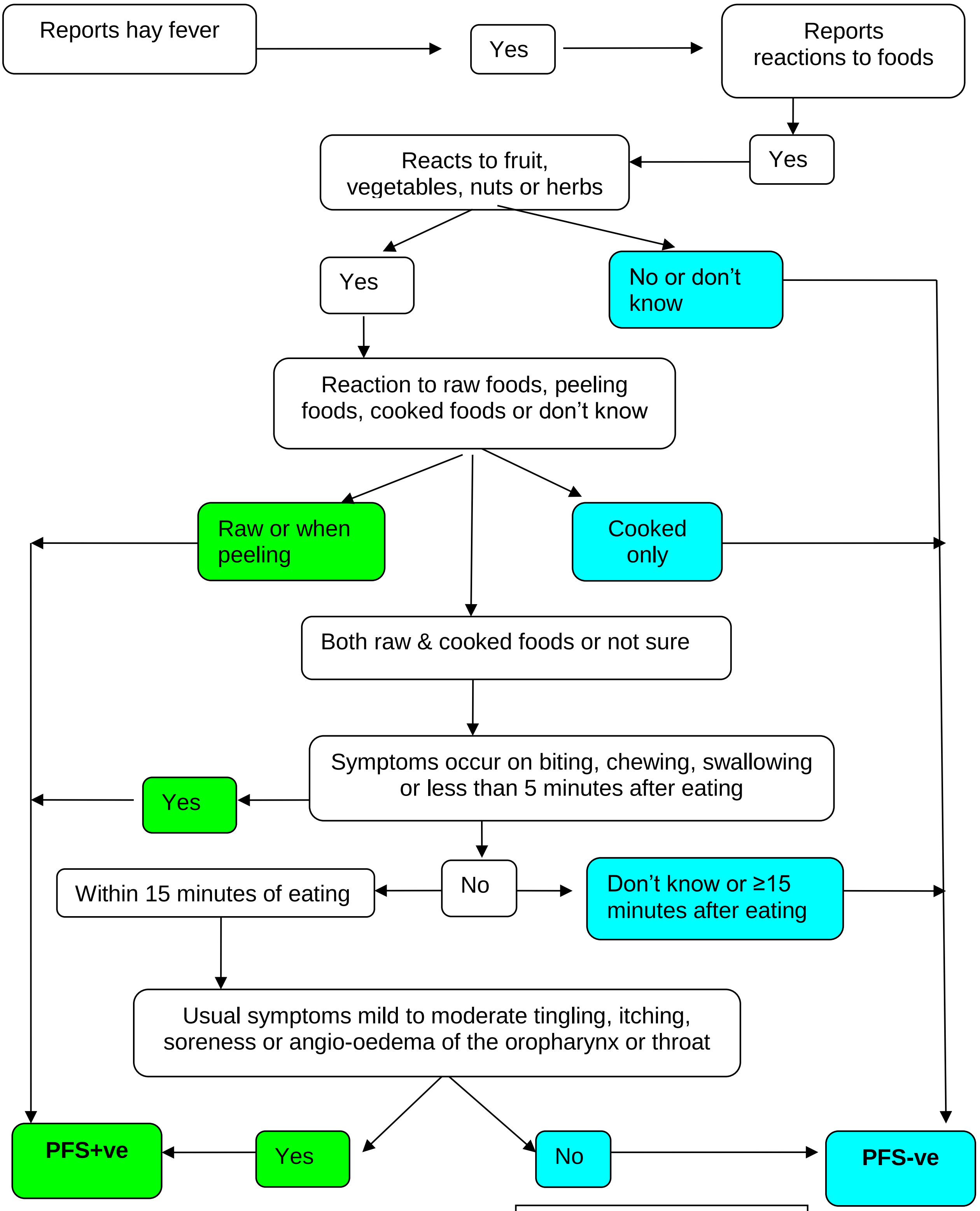

PFS+ve - has PFS

PFS-ve does not have PFS

Copyright: Royal Brompton & Harefield NHS Foundation Trust

Tests and Diagnosis

Test algorithm
Allergy and nutritional care plan

Allergy and Nutritional Care Plan

**PROVISIONAL DIAGNOSIS:**	**IgE-mediated FA** **Non-IgE-mediated FA**<br>**Other:………….** **(Circle as applicable)**
**PROVOKING FOODS**	
**OTHER CROSS-REACTING FOODS**	
**DIAGNOSTIC TESTS[1]**	
**MANAGEMENT PLAN**	
**DIETARY ADVICE**	

References

1. Muraro A, Werfel T, Hoffmann-Sommergruber K, Roberts G, Beyer K, Bindslev-Jensen C, Cardona V, Dubois A, duToit G, Eigenmann P, Fernandez Rivas M, Halken S, Hickstein L, Høst A, Knol E, Lack G, Marchisotto MJ, Niggemann B, Nwaru BI, Papadopoulos NG, Poulsen LK, Santos AF, Skypala I, Schoepfer A, Van Ree R, Venter C, Worm M, Vlieg-Boerstra B, Panesar S, de Silva D, Soares-Weiser K, Sheikh A, Ballmer-Weber BK, Nilsson C, de Jong NW, Akdis CA, EAACI Food Allergy and Anaphylaxis Guidelines Group. EAACI food allergy and anaphylaxis guidelines: diagnosis and management of food allergy. *Allergy*. 2014, 69:1008-1025.
2. Flammarion S, Santos C, Guimber D, Jouannic L, Thumerelle C, Gottrand F, Deschildre A. Diet and nutritional status of children with food allergies. Pediatr Allergy Immunol 2011, 22:161-165.
3. Boyce JA, Assa'ad A, Burks WA, Jones SM, Sampson HA, Wood RA, Plaut M, Cooper SF, Fenton MJ, Arshad SH, Bahna SL, Beck LA, Byrd-Bredbenner C, Carnargo CA, Eichenfield L, Furuta GT, Hanifin JM, Jones C, Kraft M, levy BD, Lieberman P, Luccioli S, McCall KM, Schneider LC, Simon RA, Simons FER, Teach SJ, Yawn BP, Schwaninger JM. Guidelines for the Diagnosis and Management of Food Allergy in the United States: Summary of the NIAID-Sponsored Expert Panel Report. *J Allergy ClinImmunol.* 2010;126:S1-S58.
4. Sicherer SH and Sampson HA. Food Allergy: Epidemiology, pathogenesis, diagnosis and treatment. *J Allergy Clin Immunol* 2014;133:291-307..
5. Simons FE, Ardusso LR, Bilò MB, Dimov V, Ebisawa M, El-Gamal YM, Ledford DK, Lockey RF, Ring J, Sanchez-Borges M, Senna GE, Sheikh A, Thong BY, Worm M; World Allergy organisation. World Allergy Organization Guidelines for the assessment and management of anaphylaxis. *J Allergy Clin Immunol.* 2011;127:587-593
6. Simons FE, Ardusso LR, Bilò MB, Dimov V, Ebisawa M, El-Gamal YM, Ledford DK, Lockey RF, Ring J, Sanchez-Borges M, Senna GE, Sheikh A, Thong BY, Worm M; World Allergy organisation. 2012 Update: World Allergy Organization Guidelines for the assessment and management of anaphylaxis. *Curr Opin Allergy Clin Immunol.* 2012;12:389-399
7. Gowland MH, Pumphrey RS. Further fatal allergic reactions to food in the United Kingdom, 1999-2006. J Allergy Clin Immunol 2007;119:1018-1019.
8. Cardona V, Luengo O, Garriga T, Labrador-Horrillo M, Sala-Cunill A, Izquierdo A, Soto L, Guilarte M. Co-factor-enhanced food allergy. *Allergy.* 2012;67:1316-8
9. Hompes S, Dölle S, Grünhagen J, Grabenhenrich L, Worm M. Elicitors and co-factors in food-induced anaphylaxis in adults. Clin Transl Allergy. 2013;3(1):38.
10. Niggemann B, Schmitz R, Schlaud M The high prevalence of peanut sensitization in childhood is due to cross-reactivity to pollen. *Allergy.* 2011;66:980-981
11. National Institute for Health and Clinical Excellence. Atopic Eczema in Children (CG57). http://guidance.nice.org.uk/CG57 . 2007.
12. Meyer R, Venter C, Fox AT, Shah N. Practical Dietary Management of Protein Energy Malnutrition in Young Children with Cow's Milk Protein Allergy. *Pediatr Allergy Immunol* 2012;Accepted.
13. World Health Organization, UNICEF. WHO child growth standards and the identification of severe actue malnutrition in infants and children. WS103, 1-12. 2009
14. Tuula H. Vesa, PhD, Philippe Marteau, PhD, MD and Riitta Korpela, PhD Lactose Intolerance Journal of the American College of Nutrition, 2002;19:165S-175S

15. Kneepkens CM, Hoekstra JH. Malabsorption of carbohydrates. Nestle Nutr Workshop Ser Pediatr Program 2005;56:57-69.
16. Skypala IJ and Venter C. Food Hypersensitivity (2009) Oxford. Wiley Blackwell
17. Skypala IJ, Calderon MA, Leeds AR, Emery P, Till SJ, Durham SR. Development and validation of a structured questionnaire for the diagnosis of Oral Allergy Syndrome in subjects with seasonal allergic rhinitis during the UK birch pollen season. *Clin Exp. Allergy*. 2011;41:1001-1011.
18. Dalal I, Goldberg M, Katz Y. Sesame seed food allergy. Curr Allergy Asthma Rep. 2012 Aug;12(4):339-45.
19. Morisset M ,Moneret-Vautrin D-A ,Maadi F, Fremont S, Gunard L,, Croizier A, Kanny G. Prospective study of mustard allergy: first study with double-blind placebo-controlled food challenge trials (24 cases) Allergy. 2003;58:295–299
20. Verma AK, Kumar S, Das M, Dwivedi PD.A comprehensive review of legume allergy. Clin Rev Allergy Immunol. 2013;45:30-46
21. Sanchez-Monge R, Lopez-Torrejón G, Pascual CY, Varela J, Martin-Esteban M, Salcedo G. Vicilin and convicilin are potential major allergens from pea. Clin Exp Allergy. 2004;34:1747-1753
22. Papanikolaou I, Stenger R, Bessot JC, de Blay F, Pauli G. Anaphylactic shock to guar gum (food additive E412) contained in a meal substitute. Allergy. 2007;62:822
23. Cabanillas B, Cheng H, Grimm CC, Hurlburt BK, Rodríguez J, Crespo JF, Maleki SJ. Pine nut allergy: clinical features and major allergens characterization. Mol Nutr Food Res. 2012;56:1884-1893
24. Wagner S, Breiteneder H. The latex-fruit syndrome. *Biochem Soc Tans.* 2002;30:935-940
25. Veiths S. Scheurer S and Ballmer-Weber B. Current Understanding of Cross-reactivity of food allergens and pollen. Ann N.Y. Acad. Sci. 2002; 964:47-68
26. Enrique E, Cisteró-Bahima A, Bartolomé B, Alonso R, San-Miguel-Moncin MM, Bartra J, Martinez A. Platanus acerifolia pollinosis and food allergy. Allergy 2002;75(4):351-356
27. Goetz DW, Whisman BA, Goetz AD. Cross-reactivity among edible nuts: double immunodiffusion, crossed immunoelectrophoresis, and human specific IgE serologic surveys. Ann Allergy Asthma Immunol. 2005;95:45-52.
28. Glaspole IN, de Leon MP, Prickett SR, O'Hehir RE, Rolland JM. Clinical allergy to hazelnut and peanut: identification of T cell cross-reactive allergens. Int Arch Allergy Immunol. 2011;155:345-354
29. Maleki SJ, Teuber SS, Cheng H, Chen D, Comstock SS, Ruan S, Schein CH. Computationally predicted IgE epitopes of walnut allergens contribute to cross-reactivity with peanuts. Allergy. 2011;66:1522-1529.
30. Stutius LM, Sheehan WJ, Rangsithienchai P, Bharmanee A, Scott JE, Young MC, Dioun AF, Schneider LC, Phipatanakul W. Characterizing the relationship between sesame, coconut, and nut allergy in children. Pediatr Allergy Immunol. 2010;21:1114-1118
31. Barre A, Sordet C, Culerrier R, Rancé F, Didier A, Rougé P. Vicilin allergens of peanut and tree nuts (walnut, hazelnut and cashew nut) share structurally related IgE-binding epitopes. Mol Immunol. 2008;45:1231-1240.
32. de Leon MP, Drew AC, Glaspole IN, Suphioglu C, O'Hehir RE, Rolland JM. IgE cross-reactivity between the major peanut allergen Ara h 2 and tree nut allergens. Mol Immunol. 2007;44:463-471

33. Sesame seeds and walnuts Wallowitz ML, Chen RJ, Tzen JT, Teuber SS.Ses i 6, the sesame 11S globulin, can activate basophils and shows cross-reactivity with walnut in vitro. Clin Exp Allergy. 2007;37:929-938.
34. Palacín A, Gómez-Casado C, Rivas LA, Aguirre J, Tordesillas L, Bartra J, Blanco C, Carrillo T, Cuesta-Herranz J, de Frutos C, Alvarez-Eire GG, Fernández FJ, Gamboa P, Muñoz R, Sánchez-Monge R, Sirvent S, Torres MJ, Varela-Losada S, Rodríguez R, Parro V, Blanca M, Salcedo G, Díaz-Perales A. Graph based study of allergen cross-reactivity of plant lipid transfer proteins (LTPs) using microarray in a multicenter study. PLoS One. 2012;7(12):e50799.
35. Schulten V, Nagl B, Scala E, Bernardi ML, Mari A, Ciardiello MA, Lauer I, Scheurer S, Briza P, Jürets A, Ferreira F, Jahn-Schmid B, Fischer GF, Bohle B. Pru p 3, the nonspecific lipid transfer protein from peach, dominates the immune response to its homolog in hazelnut. Allergy. 2011 Aug;66(8):1005-13.
36. Lauer I, Dueringer N, Pokoj S, Rehm S, Zoccatelli G, Reese G, Miguel-Moncin MS, Cistero-Bahima A, Enrique E, Lidholm J, Vieths S, Scheurer S. The non-specific lipid transfer protein, Ara h 9, is an important allergen in peanut. Clin Exp Allergy. 2009 Sep;39(9):1427-37.
37. Carruth BR, Skinner JD: Feeding behaviors and other motor development in healthy children (2-24 months). J Am Coll Nutr 2002;21:88-96.
38. Carruth BR, Skinner JD: Mothers' sources of information about feeding their children ages 2 months to 54 months. J Nutr Educ 2001;33:143-147.
39. Coulthard H, Harris G, Emmet P: Delayed introduction of lumpy foods to children during the complementary feeding period affects child's food acceptance and feeding at 7 years of age. Matern Child Nutr 2009;5:75-85.
40. A.L. Delaney, J.C. Arvedson Development of swallowing and feeding: Prenatal through first year of life. Dev Disabil Res Rev. 2008;14:105-17

Appendices

Appendix A. Task Force Membership

Name	Institution
Isabel Skypala (Chair and principal author)	Royal Brompton & Harefield NHS Foundation Trust, London UK
Carina Venter	The David Hide Asthma and Allergy Research Centre, Isle of Wight, UK
Rosan Meyer	Great Ormond Street NHS Foundation Trust, London, UK
Nicolette de Jong	Erasmus MC, Dept of Internal Medicine, Section Allergology, Rotterdam, Netherlands
Adam Fox	Guy’s and St Thomas’ Hospitals NHS Foundation Trust and Kings College, London, UK
Marion Groetch	Jaffe Research Institute, Mount Sinai School of Medicine, New York, USA
Hanneke Oude Elberink	University Medical Centre of Groningen, University of Groningen, Groningen, Netherlands
Aline Sprikkelman	Emma Children's Hospital Academic Medical Centre, Amsterdam, NL
Louiza Diamandi	University of Athens, Athens, Greece
Berber Vlieg-Boerstra	Emma Children's Hospital, Academic Medical Centre, Amsterdam, and Erasmus MC, Dept of Internal Medicine Netherlands
