# Supplementary material for: The development of a standardised diet history tool to support the diagnosis of food allergy
Source: Clin Transl Allergy. 2015 Feb 19;5:7. doi: 10.1186/s13601-015-0050-2 (PMC4349299; doi:10.1186/s13601-015-0050-2)
Supplement: Additional file 2: — Allergy-Focussed Diet History Adult version. [file 13601_2015_50_MOESM2_ESM.pdf]

Allergy-Focussed Diet History

Adult version

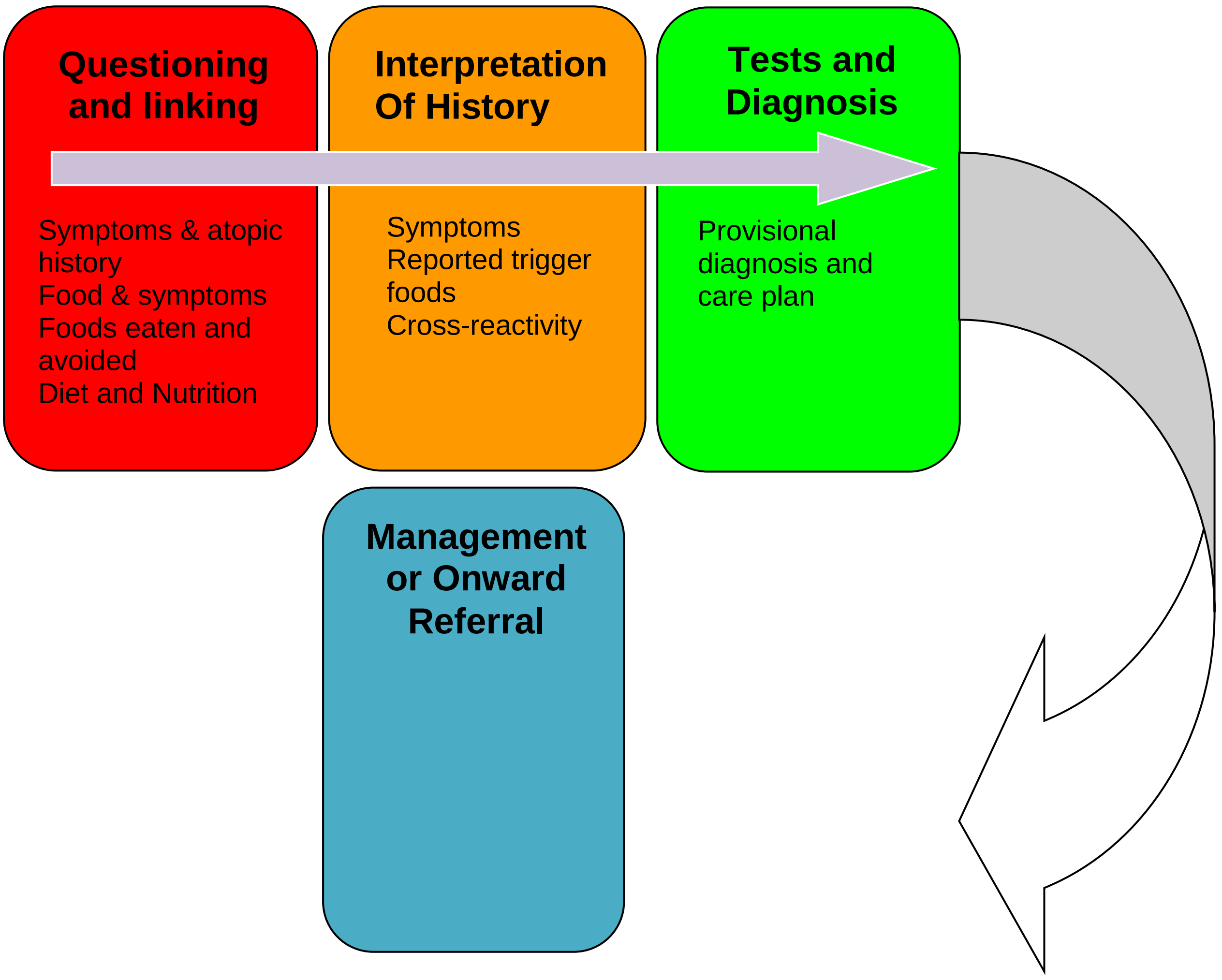

Contents

	Page
**Introduction**	3
How to use this tool	3
Defining the terms	3
Abbreviations	3
**Questioning and linking**	4
Symptom and Atopic history	4
Food and Symptoms	7
Foods eaten and avoided	9
Diet and Nutrition	10
**Interpretation of History**	11
Interpretation of symptoms	11
Interpretation of reported trigger foods	12
Linking foods eaten to specific allergens	14
Linking cross-reactivity foods and aeroallergens	17
**Tests and Diagnosis**	18
Pollen-food syndrome (Oral allergy syndrome) Algorithm	18
Allergy and nutritional care plan	19
**References**	20
**Appendices**	24
A. Task Force Membership	24

Introduction

The European Academy of Allergy and Clinical Immunology (EAACI) Guidelines on food allergy (FA)[1] suggest that the allergy-focussed history is fundamental to the establishment of the likelihood of a diagnosis, and the mechanisms and food triggers involved. In recognition of this, the Allied Health Interest group of the European Academy of Allergy and Clinical Immunology (EAACI) established a Task force (see Appendix A) to develop allergy-focussed diet history tools to facilitate a systematic standard approach to the diagnosis of adults with suspected food allergy, and support best practice and the development of pathways of care.

How to use this tool

This tool has been designed to support food allergy diagnostic pathways and determine when onward referral and/or specialist dietetic or nutritional intervention is required. A systematic approach is especially important in adults; many will be sensitised to aeroallergens, therefore testing without first taking an allergy-focussed history may confound the diagnosis rather than support it [1,2]. Careful elucidation of the symptoms and foods involved will enable a bespoke plan for further testing to be formulated. This tool is not profession-specific and can be used in its entirety by those less skilled in allergy diagnosis, or as an aide-memoir for those currently working in the allergy specialist field. It can also be utilised as an educational tool when teaching health care practitioners about food allergy.

Defining the terms

Atopy	A personal and/or familial tendency, usually in childhood or adolescence, to become sensitised and produce IgE antibodies in response to ordinary exposure to allergens, usually proteins.
Food Allergy (FA)	An adverse reaction to food mediated by an immunological mechanism, involving specific IgE (IgE mediated), cell–mediated mechanisms (non IgE mediated) or both IgE and cell mediated mechanisms (mixed IgE and non IgE mediated).
. IgE-mediated FA	Immunoglobulin E mediated and is thought to manifest as a phenotypical expression of atopy, together with (or in the absence of) atopic eczema, allergic rhinitis and/or asthma.
Non-IgE mediated FA	Cell-mediated allergy and presents mainly with gastro-intestinal symptoms in reaction to the ingestion of a food allergen.

Abbreviations

FA	Food Allergy
FHS	Food Hypersensitivity
PFS	Pollen-food Syndrome
IBS	Irritable bowel syndrome
FODMAP™	Fermentable, Oligo-, Di-, Mono-saccharides and Polyols

Questioning and linking

Symptom & atopic history
Food & symptoms
Foods eaten and avoided
Diet and Nutrition

Symptom and Atopic history

*Indicates need for onward referral for specialist allergy and/or dietary assessment

**1**	**Name**____<br><br>**Age** __________ **Male/Female** **Height**_______________**Weight**______________
**2**	**Presenting symptoms**[1,3,4] and pattern of appearance (Tick box and circle relevant symptoms)<br>☐ **Skin**: flushing/erythema, pruritus, urticaria*, angio-oedema*, eczema*<br>**Pattern** - Intermittent (on one occasion or weekly, monthly, annually) OR Continuous<br>☐ **Oro-pharyngeal –** pruritus, oedema (lips, tongue, pharynx), vocal changes (laryngeal oedema)*, throat closure*<br>**Pattern** - Intermittent (on one occasion or weekly, monthly, annually) OR Continuous<br>☐ **Gastro-intestinal**:, Abdominal pain, nausea, vomiting, diarrhoea, blood in the stool*, gastro-oesophageal reflux or dysphagia<br>**Pattern** - Intermittent (on one occasion or weekly, monthly, annually) OR Continuous<br>☐ **Upper and lower airway**: conjunctivitis, nasal itching, sneezing, rhinorrhoea with or without conjunctivitis, cough, chest tightness*, wheeze*, shortness of breath*, stridor*<br>**Pattern** - Intermittent (on one occasion or weekly, monthly, annually) OR Continuous<br>☐ ***Cardiovascular** – dizziness, hypotension, tachycardia, hypotonia (collapse)<br>**Pattern** - Intermittent (on one occasion or weekly, monthly, annually) OR Continuous<br>☐ ***Anaphylaxis** [5,6] – multi-system involvement e.g. skin symptoms plus respiratory or cardiovascular symptom, or two or more symptoms from different symptom categories<br>☐ **Other -** Pallor, tiredness, malnutrition* or other condition:<br>____<br><br>**Pattern** - Intermittent (on one occasion or weekly, monthly, annually) OR Continuous
**3**	At what age did the symptoms first appear? ________________________<br><br>In what circumstances did symptoms first appear? (During or following a meal, location etc.)<br><br>____

**4**	Where do the reactions normally take place:<br><br>Home workplace restaurant* takeaway* other location
**5**	Has any treatment/medication been taken – Yes No<br><br>If yes - what was it and did it help
**6**	Are any extrinsic factors involved[7,87] : Yes* No<br><br>If Yes – which ones:<br><br>exercise alcohol aspirin or NSAIDS fatigue/stress hormonal infection
**7**	Current and/or previous concomitant conditions:<br><br>☐ <u>Asthma[9] </u>*<br>Age onset…………….<br>On-going problem: : Yes No<br><br>Medication …………………. Symptoms: mild/moderate/severe<br><br>☐ <u>Allergic rhinitis[10]</u><br>Age onset………………….. On-going problem: Yes No<br><br>Seasonal (if yes please circle which ones)<br><br>Spring Summer Autumn OR Perennial ______________<br><br>Medication……………………… Symptoms: mild/moderate/severe<br><br>☐ <u>Food allergy in childhood</u><br>Age onset………………….<br><br>Age of resolution……………….<br><br>☐ <u>Eczema</u>* (depending on severity)<br><br>Age at onset…………………….. On-going problem Yes No<br><br>Medication………………………….. Symptoms: mild/moderate/severe<br><br>☐ <u>Other co-morbidity or chronic medical condition</u>* (depending on severity and type)

	Yes No<br><br>If yes please describe____
**8**	Family history of atopy: (if yes please circle which ones)<br><br>Asthma allergic rhinitis food allergy eczema
**9**	Any reported symptoms to latex[11] (e.g. rubber gloves, condoms or blowing up balloons)<br><br>Yes* No
**10**	Is the patient known to be sensitised to or reports previous positive tests to;<br><br>☐ Aeroallergens (if yes please circle which ones)<br><br>Grass Trees Weeds Animal dander House Dust Mite Cockroaches Moulds<br><br>Other ____<br><br>☐ Foods (if yes please circle which ones)<br><br>Milk Egg Fish Shellfish Wheat Nuts Peanuts Seeds<br><br>Other____
**10**	Is the patient:<br><br>Pregnant * Lactating Planning to conceive Not applicable
**11**	Is the patient highly anxious about their condition: Yes* No
**13**	Is the patient taking any regular medication Yes* No<br><br>If Yes please list ____

For interpretation of symptoms: see page 11

Questioning and linking

Symptom & atopic history
Food & symptoms
Foods eaten and avoided
Diet and Nutrition

Linking foods to symptoms

1	Have foods been eliminated previously Yes No<br><br>If so was this helpful? Yes No
2	Are symptoms related to food? Yes No Not sure<br><br>(If no or not sure complete Q3, if yes go to Q4 on next page)
3	If no specific food identified, list the meals preceding the most recent reaction and two other reactions including the most severe

Meal	Symptoms	Time to onset of symptoms

4	Are any foods identified which provoke symptoms? – (consider botanically related[a] or cross-reacting[b] allergenic foods)				
Food	Symptom type	Speed of onset[a]	Amount provoking reaction[b]	Raw or cooked	Symptoms every time food is eaten

(a) How many minutes or hours after eating did symptoms appear

(b) How much food provokes a reaction e.g. touching lips/inhalation* a mouthful* a few bites whole meal or snack several days of consuming the food

For interpretation of Trigger foods, see page 12

For cross-reacting foods, see page 17

Questioning and linking

Symptom & atopic history
Food & symptoms
Foods eaten and avoided
Diet and Nutrition

Foods currently being eaten and avoided

	Avoided	Eaten
**Milk - c**ow, sheep or goats milk, cheese, yoghurt,		
**Egg -**hen, duck, quail		
**Tree nuts -** Hazelnut, almond, brazil nut, walnut, pecan, cashew, pistachio, macadamia		
**Peanuts and Legumes -** soy, chickpeas, peas, beans, lentils, lupin		
**Seeds** - sesame, sunflower, pumpkin, poppy, mustard, pine nuts		
**Fresh fruit, juice or smoothies** e.g. apple, kiwifruit, peach, strawberry, banana, mango, avocado		
**Fresh vegetables and vegetable juices** e.g. tomato, carrot, celery		
**Herbs and spices** e.g. coriander, parsley, chilli, cumin, paprika, mustard		
**Cereals -** Wheat, rice, barley, oats, corn, buckwheat (not a cereal), rye, spelt, quinoa		
**Fish** (white or oily) e.g. cod, salmon, trout		
**Shellfish –** e.g. Prawns, mussels, squid		
**Meat , poultry and game**		
**Beverages -** Cordial/squash, fizzy drinks, alcohol		
**Not certain or multiple foods -** Compare the daily diet to that in Appendix C to ascertain which allergens might be involved		

For interpretation of Trigger foods, see page 12

Diet and Nutrition

*****Onward referral for nutritional assessment and intervention required

**1**	Is the patient at nutritional risk? Yes***** No<br><br>People at nutritional risk will have one of the following[12,13]:<br>a) A Body mass index (BMI) [kg/m^2] < 18.5kg/m or less<br>b) An unintentional weight loss > 10% in the last 3-6 months<br>c) A BMI < 20 and an unintentional weight loss > 5% in the last 3-6 months<br>d) A BMI > 24 or more
**2**	Are multiple foods being avoided Yes***** No
**3**	Has one or more foods been excluded for 6 months or longer?<br><br>Yes***** No
**4**	Is the patient vegetarian or vegan? Yes***** No
**5**	Any nutritional supplements taken Yes No<br><br>If yes – which supplements are taken:<br><br>____
**6**	Any religious or cultural factors that can affect food intake Yes***** No
**7**	Does the patient have a long standing chronic condition<br><br>Yes***** No<br>If Yes, what is causing the condition?...
**8**	Are deficiencies, food intolerances or accuracy of reported avoidance suspected?<br><br>Yes****** No<br><br>******Requires 24-hour diet history

Interpretation of History

Symptoms
Reported trigger foods
Cross-reactivity

Interpretation of Symptoms

SYMPTOM TYPE SEVERITY & SPEED OF ONSET		Likely IgE-mediated FA [132]	Likely non-IgE-mediated FA	Other adverse food reaction or Differential Diagnosis
SYMPTOM TYPE	Skin	Pruritus -extremities and groin<br>Urticarial rash of 6-12 hours duration<br>Acute rapid-onset urticarial/angio-oedema after eating<br>(Angio-oedema several hours after eating can occur in exercise-related FA[7] or red meat allergy due to tick bites[14])	Atopic dermatitis	Non-specific pruritus<br>Urticarial rash of more than 48 hours duration<br>Angio-oedema many hours after eating
	Gastro-intestinal	Oro-pharyngeal pruritus, oedema, tingling, paraesthesia and dysesthesia<br>Severe, acute intermittent vomiting and/or diarrhoea within 30 minutes of eating	Reproducible vomiting and/or diarrhoea to a specific food<br>Dysphagia<br>Upper GI pain (abdominal pain)	Chronic constipation or diarrhoea<br>Bloating<br>Acute lower GI pain
	Respiratory	Rhinitis, conjunctivitis, dyspnoea, wheeze, stridor, Difficulty in breathing,		Increase in mucous secretions<br>Wheeze
	Circulatory	Tachycardia or Hypotension		Hypertension
SPEED OF ONSET		IMMEDIATE<br>Reactions occur up to 2 hours after eating	INTERMEDIATE<br>Reactions occur more than 2 hours after eating	DELAYED<br>Reactions occur more than 24 hours after eating
SEVERITY		Anaphylaxis, difficulty in breathing		
REPRODUCIBILITY		Symptoms every time same food eaten		No discernible pattern

Interpretation of Reported Trigger Foods

Reported Food	Likely IgE-mediated FHS	Likely non-allergic FHS (Differential Diagnosis)
**Milk**	All forms of milk (milk, cheese, yoghurt, butter, cream) (rare in adults[15])	Milk and soft cheese (Lactose intolerance[16])<br>Milk and hard cheese (IBS[17,18])<br>Blue, strong and fermented cheese (Vaso active amines**[19,20])
**Egg**	Egg - (rare in adults)[21]	Egg
**Tree nuts and legumes**<br>NB Apply PFS algorithm[22] (page 19)	Tree nuts, peanuts, soy, other legumes (lupin, lentils, chickpeas), seeds (sesame, sunflower, pumpkin, mustard, pine nuts, poppy)	Soy
**Fresh fruit**<br>NB Apply PFS algorithm[22] (page 19)	Any fresh fruit especially apples, strawberries, plums, cherries, peaches, pears, kiwi<br>Candied peel<br>Fresh fruit juice	Dried apricots, sultanas (Sulphite hypersensitivity[23,24])<br>Unspecified acidic fruits (IBS[17])<br>Fresh fruits – especially apples and pears (IBS[17] – FODMAP® [25] foods)
**Fresh vegetables**<br>NB Apply PFS algorithm[22] (page 19)	Any fresh vegetable, especially carrots, peppers, celery (raw. cooked and spice), fresh tomatoes, sun dried tomatoes, lettuce, avocado, new potatoes in skins or jacket potatoes<br>Peeling potatoes (itchy hands)<br>Fresh herbs, especially coriander and parsley, mustard, chilli, paprika, celery salt, curry powder	Dried onions, frozen potatoes (sulphite hypersensitivity[23,24])<br>Leeks, onions, cauliflower, mushrooms, cucumber (IBS FODMAP® foods[17,24])<br>Tomato puree – histamines (Vaso active amines**[19,20])<br>Dried herbs, spices and black pepper – (salicylates**[26-29])
**Cereals**	Rice, buckwheat<br>Barley – e.g. beer, bread, malt<br>Corn – e.g. polenta, cornflakes, tortilla	Wheat or gluten (IBS – FODMAP® foods[18,25])
**Seafood**	Finned Fish (cod, herring, mackerel, sea bass, plaice etc.)<br>Crustaceans (prawns, crab, lobster, Gamba, langoustine<br>Molluscs (mussels, clams, oysters	Tuna, mackerel, swordfish, marlin, other brown fish or stale fish - Scombroid poisoning[22]<br>Poisoning from seafood toxins<br>Frozen prawns - (sulphites[23,24])
**Meat**	Beef, lamb, pork – delayed onset	Sausages – (sulphites[23,24])

	severe anaphylaxis<br>Chicken	Pepperoni sausage and other fermented sausage – (Vaso active amines**[19,20])
**Meat substitutes**	Mycoprotein (egg)<br>Vegetarian sausages, burgers or patties (soy, other legumes, nuts)	
**Beverages**	Any wine[30,31]<br>Bitter beer[33-35]<br>Wheat beer[35]	Lime cordial, bottled lemon/lime juice, cider, white wine, some lagers (sulphites[23,24])<br>red wine (Vaso active amines**[19,20])
**Composite meals or snacks**	Meals containing nuts, legumes, seeds and seafood (UK - Indian curry, Thai, Malaysian, Chinese), Hummus, Pesto (might also be in pizza or pasta dishes)<br>Other composite foods containing legumes, mustard, celery or natural food colourings [36-45]	Pizza, Pasta, Mexican, Italian, burgers, fried chicken, fish and chips,<br>Soup gravy and sauces

****evidence base is limited and lack of agreement about relevance**

Interpretation of History

Symptoms
Reported trigger foods
Cross-reactivity

Linking foods eaten to specific allergens -

Daily food pattern and possible allergens (adapted from Food Hypersensitivity, Skypala and Venter[46])*

	Milk	Egg	Wheat	Soy Legumes	Peanut/ tree nut	Other allergens
**Breakfast**						
Breakfast cereal	✓		✓		✓	Barley
Bread, Muffin, Pastry or croissant	✓	✓	✓	✓	✓	Barley
Butter/Margarine	✓					
**Snack meal**						
Soup			✓	✓		Celery, mustard
Sandwich or roll	✓	✓	✓	✓	✓	Seeds
Salad dressing	✓	✓	✓	✓		
Vinegar/ketchup			✓			Barley
Hummus				✓	✓	Sesame
Pesto	✓				✓	
Crisps	✓					
Biscuits/cake	✓	✓	✓	✓	✓	
**Main meal**						
Fish in batter		✓	✓			
Sausages		✓	✓	✓		Sulphites
Thai, Malaysian or Chinese Food		✓	✓	✓	✓	Sesame, seafood, buckwheat,
Curry	✓		✓	✓	✓	Coriander cumin, turmeric, fenugreek, celery, mustard
Pizza	✓	✓	✓	✓		Celery, mustard
Fruit tart/fritters			✓	✓		Lupin
Yoghurt/ice cream	✓	✓			✓	
**Other**						
Gluten-free						Lupin
Alcoholic drinks			✓			Grapes, barley, sulphites

***This table should be adapted for each country**

Interpretation of History

Symptoms
Reported trigger foods
Cross-reactivity

Linking foods eaten to specific allergens -

Foods likely to contain milk, egg, cereals, peanuts or tree nuts

	Milk	Egg	Wheat & Barley	Peanuts & Tree nuts
**Bread and breakfast cereals**	Breakfast cereals, some breads	French toast	Bread (sourdough, nan, soda), breakfast cereals, pancakes, muffins, crackers	Breakfast cereals, Peshawari nan bread, almond croissants
**Meat, fish, egg, cheese, vegetarian dishes**	Packet sliced cold meats, lasagne, sausages, foods in batter, sandwiches, quiche	Sausages, fish fingers, foods in batter/breadcrumbs	Sausages, foods in batter/breadcrumbs, pies, meat puddings, sandwiches	
**Pasta, rice, potato vegetables,**	Pasta in cheese or cream sauce, baked beans, soups,	Yorkshire pudding, egg fried rice, egg pasta, mycoprotein, bouillon	Potato cakes, couscous, pasta, spelt, baked beans	Vegetarian dishes
**Desserts, Cakes and biscuits**	Yoghurt, milk pudding, cheesecake, pancakes, custard, ice cream, mousse, Cakes, biscuits, muffins	Pancakes, meringue, soufflé, sponge, trifle, crème brule, egg custard, pastry cream, mousse, ice cream, cakes, pastries brushed with raw egg, marzipan, royal icing,	Semolina, cheesecake, tarts, sponge, crumble, pancakes, biscuits, cakes, pastries,	Pastries, nut cookies, Ice cream toppings, Bakewell tart, brownies, fruit cake, nut cookies, marzipan, hazelnut paste, halva, baklava, macaroons
**Restaurant and take away food**	Pizza, curry containing cream (e.g. korma)			Curry especially Korma sauce, Thai and Chinese food, Satay sauce, Szechwan sauce
**Sweets and snacks**	Toffee, fudge. Caramel, chocolate, crisps, flavoured snacks	Filled chocolates, cream/fondant fillings		Peanut/nut brittle, Marron glace, sugared almonds, praline, chocolates, nougat
**Condiments and spreads**	Salad dressing, butter, margarine	Salad dressing, mayonnaise, salad cream, Hollandaise sauce, lemon curd	Sauces, gravy	Peanut butter, Chocolate hazelnut spread, cold pressed walnut, almond and hazelnut oil peanut/groundnut oil
**Drinks**	Milk, Pineapple & coconut juice, latte, coffee and tea whiteners	Advocat	Beer, malt drinks	

***This table should be adapted for each country**

Interpretation of History

Symptoms
Reported trigger foods
Cross-reactivity

Linking foods eaten to specific allergens -

Foods likely to contain soy, lupin, sesame, mustard and sulphites

	Soy & Lupin	Sesame	Mustard	Celery	Sulphites
**Bread, crackers, breakfast cereals**	Bread, crackers, gluten-free products	Bread sticks, bagels, crackers, crisp bread, rice cakes			
**Meat, fish, egg, cheese, vegetarian dishes**	Tofu, tempeh, sausages, beef burgers baby food, battered food	Burger buns, falafel, samosa	Cheese sauce, Welsh Rarebit, ready meals, stews, casseroles, hot dogs	Ready meals, casseroles, stews	Dried salted fish, sausages, beef burgers, frozen prawns/shrimps
**Pasta, rice, potato vegetables,**	Edamame beans, noodles	Noodles	Pickled vegetables	Vegetable puree	Dried onions (e.g. on hot dogs), frozen and vacuum-packed potatoes,
**Desserts, cakes and biscuits**	Biscuits, pastries	Baklava, halva, sesame snaps, pastries,			
**Restaurant and take away food**	Pizza	Chinese and Thai food	Curry, Pizza, Mexican	Curry, pizza	
**Sweets and snacks**	Cheese dips	Hummus,			Dried fruits especially apricots, sultanas, prunes, figs, dates, bananas, Maraschino cherries
**Condiments and spreads**	Soy sauce, stock cubes, sauces, mayonnaise, salad dressing	Salad dressing, soups, sauces, dips	Salad dressing, barbeque sauce, soups, ketchup, Mayonnaise, marinades, piccalilli, chutney, curry powder	Curry powder, spice mixes, rubs, sauces, marinades, soups, salad dressing, Yeast extract, gravy, stock cubes, ketchup, Barbeque sauce, chutneys, pickles	Vinegar, horseradish sauce
**Drinks**	Soy milk, miso soup, body-building protein shakes			Vegetable juice, tomato juice, Bloody Mary	Cider, wine, lager, lime cordial, preserved lemon and lime juice, lemon cordial and other light coloured concentrated fruit drinks, grape juice, soft drinks

Interpretation of History

Symptoms
Reported trigger foods
Cross-reactivity

Linking cross-reactivity between foods and aeroallergens[22,47-58]

Primary sensitisation or allergy to:	Potential co-sensitisation or cross-reactions to:
**Pollens**	
Silver Birch	apple, pear, cherry, peach, nectarine, apricot, plum, damson, greengage, strawberry, kiwifruit, hazelnut, walnut,, almond, Brazil nut, celery, carrot, potato, soy, fig, bean sprouts, mange tout
Plane tree	hazelnut, peach, apple, melon, kiwi, peanuts, maize, chickpea, lettuce, green beans
Grass	melon, watermelon, orange, tomato, aubergine, sweet pepper, chilli or cayenne pepper, potato, peanut, Swiss chard
Mugwort	celery, celeriac, carrot, parsnip, dill, parsley, coriander, cumin, fennel, aniseed, caraway, angelica, chervil, sunflower seed, honey
Ragweed	watermelon and other melon, banana, courgette, cucumber, courgette, marrow, squash, pumpkin,
Latex	Avocado pear, chestnut, peach, banana, passion fruit, kiwi fruit, papaya, mango, tomato, pepper, potato, celery
**Foods**	
Peanut	Walnut, pistachio nut, cashew nut, Hazelnut, almond, Brazil nut, sesame seed, other legumes (see below*)
Pistachio or cashew nut	Pistachio or cashew nut, peanut, hazelnut
Walnut or pecan nut	Walnut or pecan nut, hazelnut, cashew nut, peanut, sesame
Hazelnut	Cashew, Brazil nut, pistachio, almond, peanut, walnut
Sesame	Sunflower seeds, peanuts, walnuts, hazelnut, Brazil nut, almonds
Peaches	Apples, hazelnuts, peanuts, walnuts, orange, cabbage, lettuce, mustard, corn, barley

*Legumes	Peanuts, Soy, Pigeon Pea, Goa Bean, Runner Bean, French Bean, Haricot Bean, Butter Bean, Lima Bean, Chick Pea, Mung Bean, Fava Bean, Peas, Lentils, Tamarind, Guar Gum, Fenugreek, Liquorice, Gum Arabic, Tragacanth

©Pollen-Food Syndrome (PFS) (Oral Allergy Syndrome) Algorithm[22]

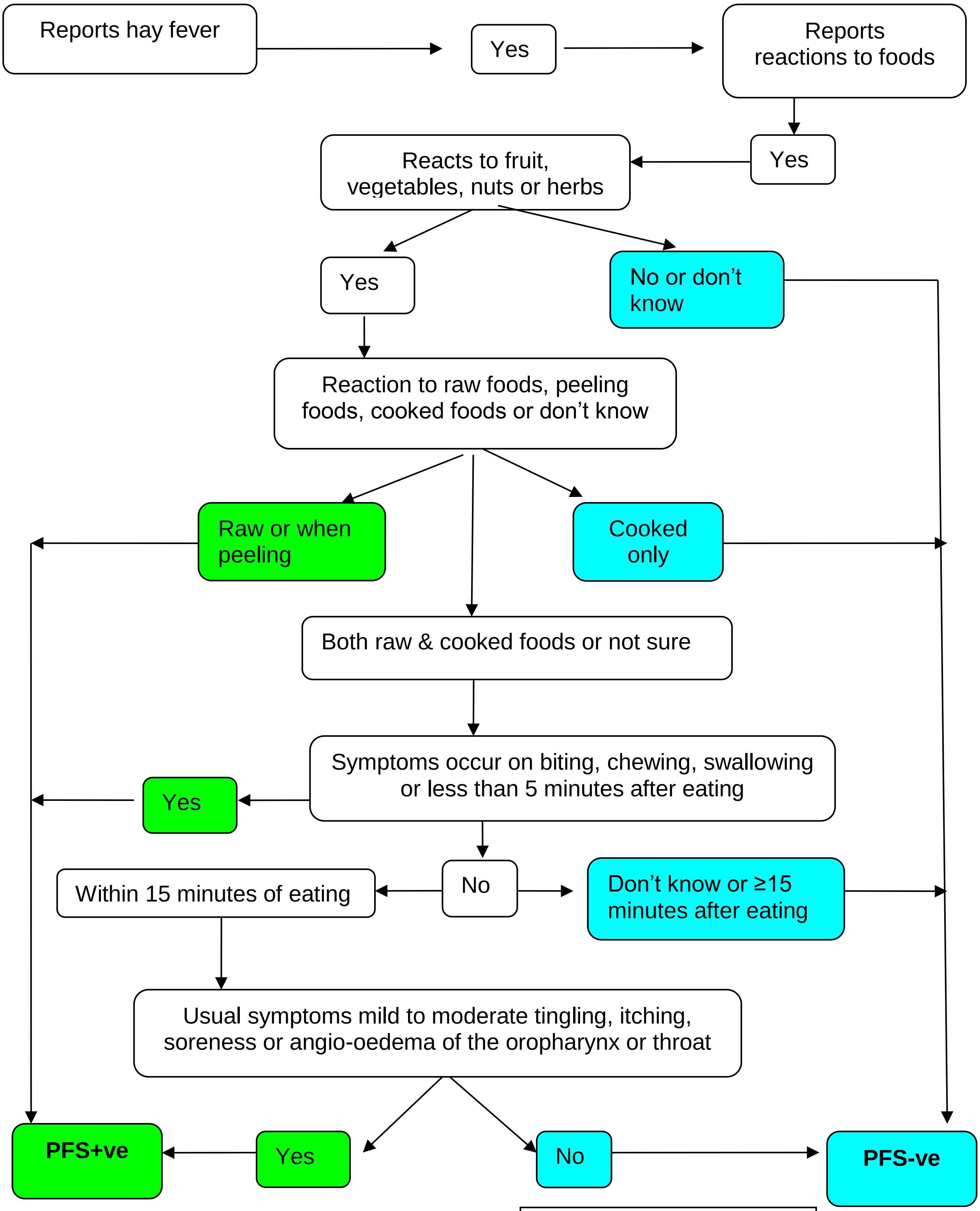

Copyright: Royal Brompton & Harefield NHS Foundation Trust

PFS+ve - has PFS

PFS-ve does not have PFS

Tests and Diagnosis

Test algorithm
Allergy and nutritional care plan

Allergy and Nutritional Care Plan

**PROVISIONAL DIAGNOSIS:**	**IgE-mediated FA** **Non-IgE-mediated FA**<br>**Other:………….** **(Circle as applicable)**
**PROVOKING FOODS**	
**OTHER CROSS-REACTING FOODS**	
**DIAGNOSTIC TESTS**[1]	
**MANAGEMENT PLAN**	
**DIETARY ADVICE**	

References

1. Muraro A, Werfel T, Hoffmann-Sommergruber K, Roberts G, Beyer K, Bindslev-Jensen C, Cardona V, Dubois A, duToit G, Eigenmann P, Fernandez Rivas M, Halken S, Hickstein L, Høst A, Knol E, Lack G, Marchisotto MJ, Niggemann B, Nwaru BI, Papadopoulos NG, Poulsen LK, Santos AF, Skypala I, Schoepfer A, Van Ree R, Venter C, Worm M, Vlieg-Boerstra B, Panesar S, de Silva D, Soares-Weiser K, Sheikh A, Ballmer-Weber BK, Nilsson C, de Jong NW, Akdis CA, EAACI Food Allergy and Anaphylaxis Guidelines Group. EAACI food allergy and anaphylaxis guidelines: diagnosis and management of food allergy. *Allergy*. 2014, 69:1008-1025.
2. Burney PG, Potts J, Kummeling I, Mills EN, Clausen M, Dubakiene R, Barreales L, Fernandez-Perez C, Fernandez-Rivas M, Le TM, Knulst AC, Kowalski ML, Lidholm J, Ballmer-Weber BK, Braun-Fahlander C, Mustakov T, Kralimarkova T, Popov T,Sakellariou A, Papadopoulos NG, Versteeg SA, Zuidmeer L, Akkerdaas JH, Hoffmann-Sommergruber K, van Ree R. The prevalence and distribution of food sensitization in European adults. Allergy. 2014;69:365-371.
3. Boyce JA, Assa'ad A, Burks WA, Jones SM, Sampson HA, Wood RA, Plaut M, Cooper SF, Fenton MJ, Arshad SH, Bahna SL, Beck LA, Byrd-Bredbenner C, Carnargo CA, Eichenfield L, Furuta GT, Hanifin JM, Jones C, Kraft M, levy BD, Lieberman P, Luccioli S, McCall KM, Schneider LC, Simon RA, Simons FER, Teach SJ, Yawn BP, Schwaninger JM. Guidelines for the Diagnosis and Management of Food Allergy in the United States: Summary of the NIAID-Sponsored Expert Panel Report. *J Allergy ClinImmunol.* 2010;126:S1-S58.
4. Sicherer SH and Sampson HA. Food Allergy: Epidemiology, pathogenesis, diagnosis and treatment. *J Allergy Clin Immunol* 2014;133:291-307..
5. Simons FE, Ardusso LR, Bilò MB, Dimov V, Ebisawa M, El-Gamal YM, Ledford DK, Lockey RF, Ring J, Sanchez-Borges M, Senna GE, Sheikh A, Thong BY, Worm M; World Allergy organisation. World Allergy Organization Guidelines for the assessment and management of anaphylaxis. *J Allergy Clin Immunol.* 2011;127:587-593
6. Simons FE, Ardusso LR, Bilò MB, Dimov V, Ebisawa M, El-Gamal YM, Ledford DK, Lockey RF, Ring J, Sanchez-Borges M, Senna GE, Sheikh A, Thong BY, Worm M; World Allergy organisation. 2012 Update: World Allergy Organization Guidelines for the assessment and management of anaphylaxis. *Curr Opin Allergy Clin Immunol.* 2012;12:389-399
7. Cardona V, Luengo O, Garriga T, Labrador-Horrillo M, Sala-Cunill A, Izquierdo A et al. Co-factor-enhanced food allergy. *Allergy.* 2012;**67**:1316-1318
8. Hompes S, Dölle S, Grünhagen J, Grabenhenrich L, Worm M. Elicitors and co-factors in food-induced anaphylaxis in adults. Clin Transl Allergy. 2013;**3**:38.
9. Gowland MH, Pumphrey RS. Further fatal allergic reactions to food in the United Kingdom, 1999-2006. J Allergy Clin Immunol 2007;119:1018-1019.
10. Schäfer T, Böhler E, Ruhdorfer S, Weigl L, Wessner D, Heinrich J, Filipiak B, Wichmann HE, Ring J. Epidemiology of food allergy/food intolerance in adults: associations with other manifestations of atopy *Allergy*. 2001;56:1172-1179
11. Wagner S, Breiteneder H. The latex-fruit syndrome. *Biochem Soc Tans.* 2002;30:935-940
12. http://www.bapen.org.uk/pdfs/must/must_full.pdf

13. National Collaborating Centre for Acute Care, February 2006. Nutrition support in adults Oral nutrition support, enteral tube feeding and parenteral nutrition. National Collaborating Centre for Acute Care, London. www.rcseng.ac.uk
14. Commins SP, James HR, Stevens W, Pochan SL, Land MH, King C et al. Delayed clinical and ex vivo response to mammalian meat in patients with IgE to galactose-alpha-1,3-galactose. J Allergy Clin Immunol. 2014 Mar 19. [Epub ahead of print]
15. Wuthrich B, Schmid A, Walther B, Seiber R. Milk consumption does not lead to mucus production or occurrence of asthma. J Am Coll Nutr 2005;24:547S-555S
16. Tuula H. Vesa, PhD, Philippe Marteau, PhD, MD and Riitta Korpela, PhD Lactose Intolerance Journal of the American College of Nutrition, 2002;19:165S-175S
17. National Institute for Health and Clinical Excellence. Irritable bowel syndrome in adults. Diagnosis and management of irritable bowel syndrome in primary care London: NICE, 2008.www.nice.org.uk/CG061 .
18. Monsbakken KW, Vandvik PO, Farup PG. Perceived food intolerance in subjects with irritable bowel syndrome – aetiology, prevalence and consequences. *Eur J Clin Nutr*. 2006;60:667-672.
19. Maintz L, Novak N. Histamine and histamine intolerance. Am J Clin Nutr 2007;85:1185−1196
20. Fritz SB, Baldwin JL. Pharmacologic food reactions. In: Food Allergy: adverse reactions to foods and food additives. Metcalfe DD, Sampson HJ, Simon RA. 2003 pp324-341. Blackwell Publishing Ltd. Oxford
21. Szépfalusi Z, Ebner C, Pandjaitana R, Orlicek F, Scheiner O, Boltz-Nitulescu G, Ebner H. Egg yolk α-livetin (chicken serum albumin) is a cross-reactive allergen in the bird-egg syndrome. *J All Clin Immunol.* 1994;93:932-942
22. Skypala IJ, Calderon MA, Leeds AR, Emery P, Till SJ, Durham SR. Development and validation of a structured questionnaire for the diagnosis of Oral Allergy Syndrome in subjects with seasonal allergic rhinitis during the UK birch pollen season. *Clin Exp. Allergy*. 2011;41:1001-1011.
23. Leclercq c, Molinaro MG, Piccinelli R, Baldini M, Arcella D and Stacchini P. Dietary intake exposure to sulphites in Italy – analytical determination of sulphite-containing foods and their combination into standard meals for adults and children. . Food Additives and Contaminants, 2000. Vol 17 No. 12, 979-989.
24. Taylor SL, Bush RK, Nordlee JA. Sulfites. In: Food Allergy: adverse reactions to foods and food additives. Metcalfe DD, Sampson HJ, Simon RA. 2003 pp324-341. Blackwell Publishing Ltd. Oxford
25. Gibson PR, Shepherd SJ. Evidence-based dietary management of functional gastrointestinal symptoms: The FODMAP approach. *J Gastroenterol Hepatol* 2010;25:252-258
26. Scotter MJ, Roberts DPT, Wilson LA, Howard FAC, Davis J and Mansell N. Free salicylic acid and acetyl salicylic acid content of foods using gas chromatography-mass spectrometry. Food Chem 2007;105:273-279
27. Swain AR, Dutton SP, Truswell AS. Salicylates in Foods. Journal of the American Dietetic Association 1985;85(8):950-960
28. Venema DP, Hollman P. C. H, Janssen KPLTM, Katan MB. Determination of acetylsalicylic acid and salicylic acid in foods, using HPLC with fluorescence detection. J Agric Food Chem 1996;44:1762-1767

29. Wood A, Baxter G, Thies F, Kyle J, Duthie G. A systematic review of salicylates in foods: Estimated daily intake of a Scottish population. Mol Nutr. Food Res. 2011:55:S7-S14
30. Kalogeromitros DC, Makris MP, Gregoriou SG, Mousatou VG, Lyris NG, Tarassi KE, Papasteriades CA.. Grape anaphylaxis: a study of 11 adult onset cases. Allergy Asthma Proc 2005:26:53-58
31. Schad SG, Trcka J, Vieths S, Scheurer S, Conti A, Brocker EB, Trautmann A. Wine anaphylaxis in a German patient: IgE−mediated allergy against a lipid transfer protein of grapes. Int Arch Allergy Immunol 2005;136:159−164
32. Curioni A, Santucci B, Cristaudo A, Canistraci C, Pietravalle M, Simonato B, Giannattasio M Urticaria from beer: an immediate hypersensitivity reaction due to a 10-kDa protein derived from barley. Clin Exp Allergy 1999;29:407-413
33. Figueredo E, Quirce S, del-Amo A, Cuesta J, Arrieta I, Lahoz C, Sastre J. Beer-induced anaphylaxis: identification of allergens. Allergy 1999;54: 630-634
34. Asero R, Mistrello G, Roncarolo D, Amato S, van-Ree R. A case of allergy to beer showing cross-reactivity between lipid transfer proteins. Ann Allergy Asthma Immunol 2001;87:65-67**)**
35. Herzinger T, Kick G, Ludolph-Hauser D, Przybilla B. Anaphylaxis to wheat beer. Ann-Allergy-Asthma-Immunol 2004;92:673-675
36. Dalal I, Goldberg M, Katz Y. Sesame seed food allergy. Curr Allergy Asthma Rep. 2012 Aug;12(4):339-45.
37. Morisset M ,Moneret-Vautrin D-A ,Maadi F, Fremont S, Gunard L,, Croizier A, Kanny G. Prospective study of mustard allergy: first study with double-blind placebo-controlled food challenge trials (24 cases) Allergy. 2003;58:295–299
38. Verma AK, Kumar S, Das M, Dwivedi PD.A comprehensive review of legume allergy. Clin Rev Allergy Immunol. 2013;45:30-46
39. Sanchez-Monge R, Lopez-Torrejón G, Pascual CY, Varela J, Martin-Esteban M, Salcedo G. Vicilin and convicilin are potential major allergens from pea. Clin Exp Allergy. 2004;34:1747-1753
40. Papanikolaou I, Stenger R, Bessot JC, de Blay F, Pauli G. Anaphylactic shock to guar gum (food additive E412) contained in a meal substitute. Allergy. 2007;62:822
41. Cabanillas B, Cheng H, Grimm CC, Hurlburt BK, Rodríguez J, Crespo JF, Maleki SJ. Pine nut allergy: clinical features and major allergens characterization. Mol Nutr Food Res. 2012;56:1884-1893
42. Ballmer-Weber BK, Hoffmann A, Wüthrich B, Lüttkopf D, Pompei C, Wangorsch A, Kästner M, Vieths S. Influence of food processing on the allergenicity of celery: DBPCFC with celery spice and cooked celery in patients with celery allergy. Allergy. 2002;57:228-235
43. Heffler E, Nebiolo F, Asero R, Guida G, Badiu I, Pizzimenti S et al. Clinical manifestations, co-sensitizations, and immunoblotting profiles of buckwheat-allergic patients. Allergy. 2011;66:264-270
44. Liippo J, Lammintausta K. Allergy to carmine red (E120) is not dependent on concurrent mite allergy. Int Arch Allergy Immunol. 2009;150:179-83
45. Ebo DG, Ingelbrecht S, Bridts CH, Stevens WJ. Allergy for cheese: evidence for an IgE-mediated reaction from the natural dye annatto. Allergy. 2009;64:1558-1560

46. Skypala IJ and Venter C. Food Hypersensitivity (2009) Oxford. Wiley Blackwell
47. Vieths S. Scheurer S and Ballmer-Weber B. Current Understanding of Cross-reactivity of food allergens and pollen. Ann N.Y. Acad. Sci. 2002; 964:47-68
48. Enrique E, Cisteró-Bahima A, Bartolomé B, Alonso R, San-Miguel-Moncin MM, Bartra J, Martinez A. Platanus acerifolia pollinosis and food allergy. Allergy 2002;75(4):351-356
49. Goetz DW, Whisman BA, Goetz AD. Cross-reactivity among edible nuts: double immunodiffusion, crossed immunoelectrophoresis, and human specific IgE serologic surveys. Ann Allergy Asthma Immunol. 2005;95:45-52.
50. Glaspole IN, de Leon MP, Prickett SR, O'Hehir RE, Rolland JM. Clinical allergy to hazelnut and peanut: identification of T cell cross-reactive allergens. Int Arch Allergy Immunol. 2011;155:345-354
51. Maleki SJ, Teuber SS, Cheng H, Chen D, Comstock SS, Ruan S, Schein CH. Computationally predicted IgE epitopes of walnut allergens contribute to cross-reactivity with peanuts. Allergy. 2011;66:1522-1529.
52. Stutius LM, Sheehan WJ, Rangsithienchai P, Bharmanee A, Scott JE, Young MC, Dioun AF, Schneider LC, Phipatanakul W. Characterizing the relationship between sesame, coconut, and nut allergy in children. Pediatr Allergy Immunol. 2010;21:1114-1118
53. Barre A, Sordet C, Culerrier R, Rancé F, Didier A, Rougé P. Vicilin allergens of peanut and tree nuts (walnut, hazelnut and cashew nut) share structurally related IgE-binding epitopes. Mol Immunol. 2008;45:1231-1240.
54. de Leon MP, Drew AC, Glaspole IN, Suphioglu C, O'Hehir RE, Rolland JM. IgE cross-reactivity between the major peanut allergen Ara h 2 and tree nut allergens. Mol Immunol. 2007;44:463-471
55. Wallowitz ML, Chen RJ, Tzen JT, Teuber SS.Ses i 6, the sesame 11S globulin, can activate basophils and shows cross-reactivity with walnut in vitro. Clin Exp Allergy. 2007;37:929-938.
56. Palacín A, Gómez-Casado C, Rivas LA, Aguirre J, Tordesillas L, Bartra J, Blanco C, Carrillo T, Cuesta-Herranz J, de Frutos C, Alvarez-Eire GG, Fernández FJ, Gamboa P, Muñoz R, Sánchez-Monge R, Sirvent S, Torres MJ, Varela-Losada S, Rodríguez R, Parro V, Blanca M, Salcedo G, Díaz-Perales A. Graph based study of allergen cross-reactivity of plant lipid transfer proteins (LTPs) using microarray in a multicenter study. PLoS One. 2012;7(12):e50799.
57. Schulten V, Nagl B, Scala E, Bernardi ML, Mari A, Ciardiello MA, Lauer I, Scheurer S, Briza P, Jürets A, Ferreira F, Jahn-Schmid B, Fischer GF, Bohle B. Pru p 3, the nonspecific lipid transfer protein from peach, dominates the immune response to its homolog in hazelnut. Allergy. 2011 Aug;66(8):1005-13.
58. Lauer I, Dueringer N, Pokoj S, Rehm S, Zoccatelli G, Reese G, Miguel-Moncin MS, Cistero-Bahima A, Enrique E, Lidholm J, Vieths S, Scheurer S. The non-specific lipid transfer protein, Ara h 9, is an important allergen in peanut. Clin Exp Allergy. 2009 Sep;39(9):1427-37.

Appendices

Appendix A. Task Force Membership

Name	Institution
Isabel Skypala (Chair and principal author)	Royal Brompton & Harefield NHS Foundation Trust, London UK
Carina Venter	The David Hide Asthma and Allergy Research Centre, Isle of Wight, UK
Rosan Meyer	Great Ormond Street NHS Foundation Trust, London, UK
Nicolette de Jong	Erasmus MC, Dept of Internal Medicine, Section Allergology, Rotterdam, Netherlands
Adam Fox	Guy's and St Thomas' Hospitals NHS Foundation Trust and Kings College, London, UK
Marion Groetch	Jaffe Research Institute, Mount Sinai School of Medicine, New York, USA
Hanneke Oude Elberink	University Medical Centre of Groningen, University of Groningen, Groningen, Netherlands
Aline Sprikkelman	Emma Children's Hospital Academic Medical Centre, Amsterdam, NL
Louiza Diamandi	University of Athens, Athens, Greece
Berber Vlieg-Boerstra	Emma Children's Hospital, Academic Medical Centre, Amsterdam, and Erasmus MC, Dept of Internal Medicine Netherlands
